# Like mother like daughter, the role of low human capital in intergenerational cycles of disadvantage: the Pune Maternal Nutrition Study

**DOI:** 10.3389/fgwh.2024.1174646

**Published:** 2025-01-20

**Authors:** Akanksha A. Marphatia, Jonathan C. K. Wells, Alice M. Reid, Aboli Bhalerao, Chittaranjan S. Yajnik

**Affiliations:** ^1^ Population, Policy and Practice Department, UCL Great Ormond Street Institute of Child Health, London, United Kingdom; ^2^ Department of Geography, University of Cambridge, Cambridge, United Kingdom; ^3^ Diabetes Unit, King Edwards Memorial Hospital Research Centre, Pune, India

**Keywords:** maternal and child undernutrition, low education, early marriage, infant and child growth, human capital, wealth, intergenerational effects, cardiometabolic risk

## Abstract

**Introduction:**

Maternal nutrition promotes maternal and child health. However, most interventions to address undernutrition are only implemented once pregnancy is known, and cannot address broader risk factors preceding conception. Poverty and socio-economic status are considered systemic risk factors, but both economic growth and cash transfers have had limited success improving undernutrition. Another generic risk factor is low human capital, referring to inadequate skills, knowledge and autonomy, and represented by traits such as low educational attainment and women's early marriage. Few studies have evaluated whether maternal human and socio-economic capital at conception are independently associated with maternal and offspring outcomes.

**Methods:**

Using data on 651 mother-child dyads from the prospective Pune Maternal Nutrition Study in rural India, composite markers were generated of “maternal human capital” using maternal marriage age and maternal and husband's education, and 'socio-economic capital' using household wealth and caste. Linear and logistic regression models investigated associations of maternal low/mid human capital, relative to high capital, with her own nutrition and offspring size at birth, postnatal growth, education, age at marriage and reproduction, and cardiometabolic risk at 18 years. Models controlled for socio-economic capital, maternal age and parity.

**Results:**

Independent of socio-economic capital, and relative to high maternal human capital, low human capital was associated with shorter maternal stature, lower adiposity and folate deficiency but higher vitamin B_12_ status. In offspring, low maternal human capital was reflected in shorter gestation, smaller birth head girth, being breastfed for longer, poor postnatal growth, less schooling, lower fat mass and insulin secretion at 18 years. Daughters married and had children at an early age.

**Discussion:**

Separating maternal human and socio-economic capital is important for identifying the aspects which are most relevant for future interventions. Low maternal human capital, independent of socio-economic capital, was a systemic risk factor contributing to an intergenerational cycle of disadvantage, perpetuated through undernutrition, low education and daughters' early marriage and reproduction. Future interventions should target maternal and child human capital. Increasing education and delaying girls' marriage may lead to sustained intergenerational improvements across Sustainable Development Goals 1 to 5, relating to poverty, hunger, health, education and gender equality.

## Introduction

Maternal undernutrition is a chronic problem associated with adverse maternal and child health outcomes, potentially leading to an intergenerational cycle of disadvantage (1–3). In low- and middle-income countries, maternal shorter stature has been associated with greater offspring mortality risk and higher prevalence of infant and child wasting (acute undernutrition, measured as low weight-for-height) and stunting (chronic undernutrition, measured as low height-for-age) (4–7). Biochemical markers of maternal undernutrition status such as anaemia also predict adverse child health outcomes, including low birthweight, preterm birth and perinatal and neonatal mortality (8). In a prospective rural Indian birth cohort, lower intake of maternal micronutrient-rich foods during pregnancy and lower red cell folate concentrations were associated with smaller birth size (9). Lower maternal vitamin B_12_ and higher folate status predicted higher child adiposity and insulin resistance, markers of cardiometabolic risk (10, 11). Similarly, offspring experiencing poor early growth have increased susceptibility to non-communicable disease in adulthood (12, 13).

Most interventions to address maternal undernutrition, such as macronutrient or micronutrient supplementation, are implemented once pregnancy status is already known (14). While individual studies have reported success in some settings (15, 16), a recent review concluded that it is still uncertain whether transient maternal micronutrient or food supplementation during pregnancy can systematically reduce the risk of low birthweight, small-for-gestational age neonates or preterm birth (17). Moreover, any short-term improvements in size at birth do not necessarily translate into long-term benefits for child health. For example, compared to maternal folic acid and iron supplementation, there is no clear evidence that multi-micronutrient supplementation improves child survival, growth, body composition, blood pressure, respiratory health or cognitive outcomes (18). Likewise, combined food and micronutrient supplementation had little benefit for childhood blood pressure in Bangladesh (19), while maternal protein/energy supplementation did not benefit the offspring's body composition or cardiometabolic health in the Gambia (20–22). One potential explanation for these results may be that short-term interventions during pregnancy do not address broader risk factors for undernutrition that are present prior to conception and persist beyond the pregnancy.

We therefore need to consider systemic risk factors that are difficult to ameliorate once pregnancy has already begun, and which may persistently impact both maternal and child outcomes. These include not only maternal pre-pregnancy nutritional status, but also longer-term developmental factors (maternal growth, stature) and/or overarching background factors (socio-economic status). These factors typically operate over an intergenerational timescale; hence, the current phenotype and status of women are likely to reflect those of their own mothers.

Poverty is often presented as the key underlying driver of maternal and child undernutrition (23–25), but there is considerable debate on the supporting evidence. Some studies have associated national economic growth (Gross Domestic Product, GDP per capita) and greater household wealth with lower prevalences of maternal underweight and childhood stunting (26–29). For example, Smith and Haddad reported strong associations between per capita national income and improvements in child nutritional status across 63 countries (30), while Soriano et al. found that faster and sustained economic growth in 22 low- and middle-income countries resulted in large annual reductions in undernutrition (31). However, others have found that GDP is associated with only a small reduction in the prevalence of low birthweight and child malnutrition (32–35), such as Vollmer et al.'s analysis of 121 countries (36). These results may be explained by the unequal distribution of economic growth in low-income countries, whereby wealth does not reach the most undernourished and poor populations (37). GDP may also not capture the social and economic factors that impact child undernutrition (38).

At a local level, conditional cash transfers given to poor households have also had inconsistent effects on improving child undernutrition (39, 40). A broader review of different financial incentives found that despite the low quality of evidence on the effectiveness of these interventions, there were indications of their potential positive impacts on receiving colostrum, early initiation of breastfeeding, exclusive breastfeeding and mean duration of exclusive breastfeeding (41). However, a cluster-randomised controlled trial in Burkina Faso found no evidence of monthly unconditional cash transfers given to poor households over two years reducing the cumulative incidence of wasting or stunting among children < 36 months (42). Like nutritional interventions, a limitation of cash-transfer programmes may be their transient nature, such that they do not expose children to sustained improvements in the quality of their environment.

### Conceptual framework: maternal capital

To improve our understanding of intergenerational cycles of undernutrition, we therefore need to evaluate a broader range of determinants that may have prolonged impact on maternal and child nutrition. An over-arching conceptual framework, used in both evolutionary biology and the social sciences, highlights a variety of forms of “capital” that individuals can acquire (Box 1).

Box 1 **Defining forms of capital.**

‘Capital’ is a generic term used to quantify attributes that can be acquired by individuals (43) and may be subdivided into a variety of different forms. From an evolutionary perspective, for example, the physical traits of an individual organism can be considered as somatic capital. Knowledge and skills relating to the brain can be considered as cognitive capital. However many other forms of capital that relate to an individual can be considered under the umbrella of the ‘extended phenotype’ (44), a conceptual approach that recognises that many factors that benefit Darwinian fitness may be external to the physical body and may be expressed cognitively, materially or socially.

In the social sciences economic capital is well established to index various aspects of material wealth accumulated by individuals or households. Similarly, human capital is often referred to as “the stock of skills and productive knowledge embodied in people” (38) which has some similarity with Bourdieu's concept of ‘cultural capital’ (45). The knowledge, training and skills that constitute human capital can be acquired from informal and formal education (46).

Collectively, the various components of human capital can be used by individuals to access a wide range of other resources such as life opportunities, material assets, financial income and health care (47). In turn, human capital may therefore be seen as a critical gateway to ‘economic capital’. However, unlike financial components of capital which can be lost as well as gained, human capital is relatively stable, allowing an individual to benefit over the long-term from acquired knowledge and skills (46).

We assume that the importance of women's ‘human capital’ in intergenerational cycles of undernutrition may derive from its central role in enhancing dimensions of capital that relate specifically to physical reproduction. Through the lens of evolutionary theory, ‘maternal capital’ is a broad term defined as “any aspect of maternal phenotype whether somatic or behavioural which enables differential investment in offspring” (48). Relevant components of maternal phenotype include physical traits such as energy stores and social traits such as a network of support that allow a mother to invest in her offspring (48). These traits are accumulated through the mother's life-course and generate long-term impacts on the developmental trajectory of her offspring (49). For example, during early ‘critical windows’ of physiological sensitivity (50) in fetal life and also to an extent in infancy, offspring are directly exposed to maternal phenotype rather than the external environment (48, 51).

Human capital may be a key determinant of maternal capital because greater education may help mothers invest their resources in offspring more effectively, while later age at marriage may affect the mother's status in the household and the schedule of producing offspring.

Differentiating the role of different types of capital in intergenerational cycles of undernutrition is important in the context of public health because we need to identify which components of capital can be targeted by interventions at particular periods of the life-course, and who should be the target of the intervention (e.g., girls and human capital, as opposed to adult men and household wealth).

**Figure 1:**
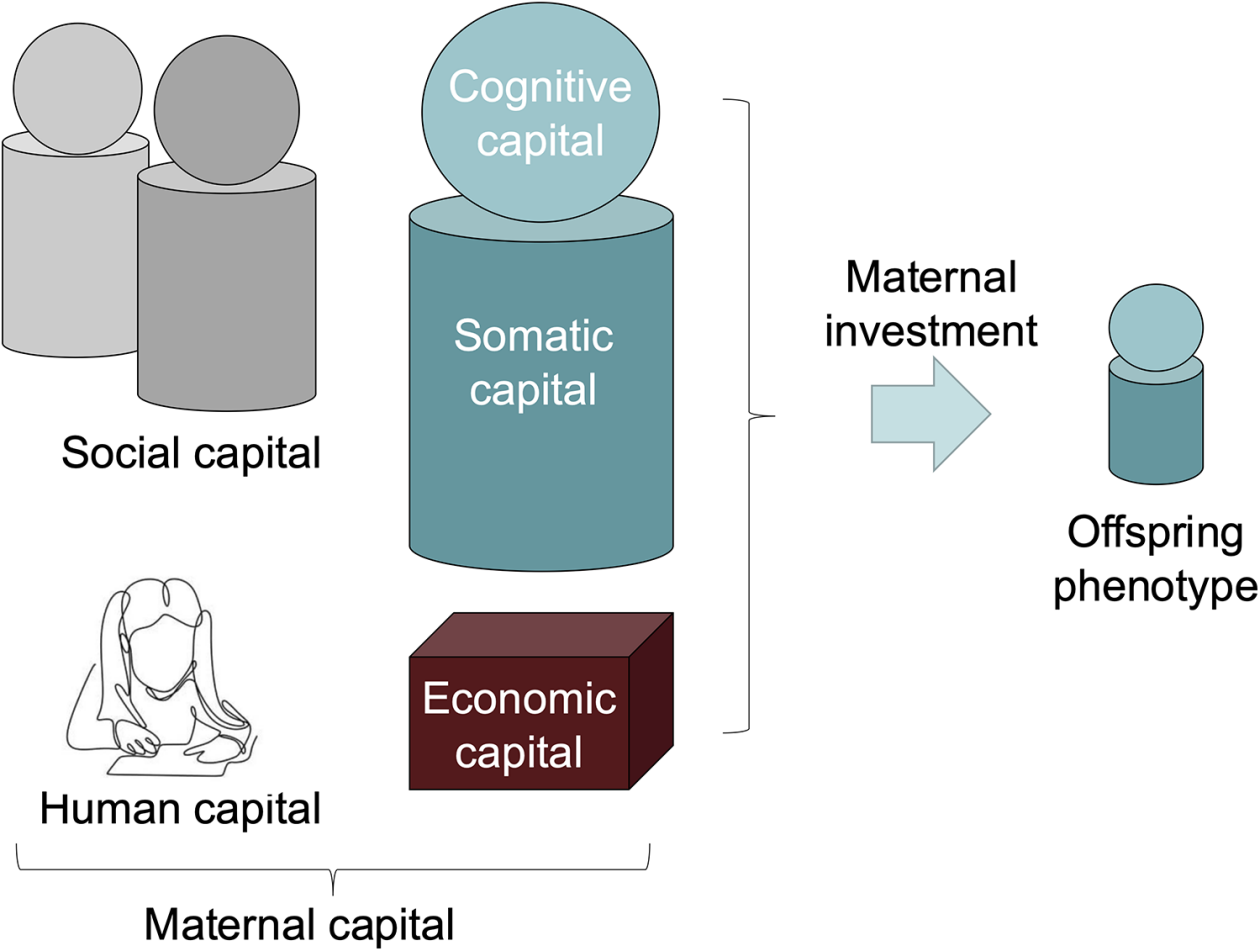
The maternal capital conceptual framework. This schematic diagram highlights different components of maternal capital, all of which promote the capacity of mothers to invest in their offspring (48). The framework draws on two key concepts from evolutionary biology, namely the extended phenotype (43) and embodied capital (42). The framework brings these together by considering how the extended phenotype of mothers includes both physical traits in the body, cognitive knowledge and capacities in the brain, and material and social assets that are external to the mother's own body, but which can promote her capacity to invest in her offspring. Somatic capital in the body refers to energy and nutrient stores, and physiological mechanisms that underpin a healthy environment for foetus during pregnancy, and a healthy supply of breastmilk during lactation. Cognitive capital refers to the capacity to implement maternal behaviours that are beneficial for the offspring, and is closely associated with human capital, referring to beneficial knowledge, skills and education gained through formal or informal means during development. In our study, we assume that early marriage may undermine human capital, by reducing the opportunity for education and/or constraining maternal autonomy. Social capital refers to a network of kin and others who provide support to the mother. Mothers may display co-variance in these different components of capital, however it is also valuable to examine their independent associations with offspring phenotype. Picture of child studying shown above human capital from (52).

Using this approach, it is possible to expand our concept of phenotype beyond the physical body, and consider a range of factors that relate to an individual through the lens of the “extended phenotype” (44). Maternal capital is a broad term that encompasses any trait that enables mothers to invest in their offspring (48). Figure 1 illustrates our conceptual approach, which focuses on different aspects of maternal capital, such as human, social, cognitive and somatic capital. What is missing from public health nutrition is a comprehensive understanding of how mothers can accumulate different forms of maternal capital through their development and reproductive career, which in turn can optimise their nutritional investment in the next generation.

The importance of maternal capital for offspring health has been shown in diverse studies, with a particular focus on biological traits. For example, across 54 low- and middle-income countries, greater maternal height (a marker of somatic capital) was associated with lower risks of mortality, wasting and stunting in the offspring (5). However, a number of recent studies have highlighted similar associations for other components of capital. For example, in the Young Lives study of families in Peru, Ethiopia, Vietnam and India, maternal social capital was associated with better infant growth (53), while analyses across low- and middle-income countries find that gender inequality (a composite national index of women's disadvantages in reproductive health, empowerment and labour market participation, relating to human capital) was more relevant than GDP per capita for low birthweight, child undernutrition (54) and child mortality (55).

However, few studies have investigated comprehensive associations of maternal capital with offspring outcomes across the full period of development. In a Brazilian birth cohort, a composite index of maternal capital reflecting maternal height, pre-pregnancy weight, education and household income was associated with diverse aspects of offspring health and human capital, including size at birth, adult height and body composition, schooling, early reproduction and risky behaviour, though associations with cardiometabolic risk were inconsistent between the sexes (56). While this study shows the powerful impact of maternal capital on offspring from infancy to adulthood across a wide range of outcomes, a limitation is that it did not disentangle individual components of maternal somatic, economic and human capital.

‘Human capital’ is a composite term used to describe knowledge, skills and autonomy (43, 46), which may be especially important for mothers. Several studies find that relative to socioeconomic status or capital, maternal nutrition and education are more important for reducing childhood undernutrition (33, 57, 58). Of particular importance for our approach, human capital refers to relatively stable components of phenotype, that can potentially benefit both a mother and her offspring throughout their life-courses.

Women's early marriage, defined by the United Nations as occurring before 18 years (59), can be considered a key determinant of low maternal human capital that is of special interest to policymakers. The early marriage of women has been associated with multiple disadvantages that may collectively drive maternal and child malnutrition, while also reflecting other depletions of human capital (60, 61). For example, women who marry early tend to have lower education (62–66) and marry into households that are economically disadvantaged, including in their food security (67). Compared to women marrying >18 years, early married women are more likely to have low access to health care (68, 69), poor nutritional status (70), shorter stature (71, 72), poor access to contraception (73), pregnancy and birth complications (74–76), shorter birth intervals (77, 78) and higher completed fertility (79, 80). These adverse associations of women's early marriage are particularly concerning because globally, 21% of women aged 20–24 years married before this age (81, 82) and in rural India, where our study is based, 27% of women were affected (83).

Unsurprisingly, therefore, women's early marriage has also been associated with adverse outcomes in the offspring, including preterm birth (84–86), smaller birth size (87), poor infant and child morbidity (88), undernutrition (87, 89–93) and infant and child mortality (94–96). Further studies have shown that women's low education, early marriage and undernutrition are associated with lower educational attainment and early marriage of their daughters (87, 97–100).

Overall, therefore, studies show that both social (low education, early marriage) and physical (reduced growth, undernutrition) traits recur across generations, suggesting that these different aspects of phenotype constitute an overarching cycle of disadvantage. Elucidating the roles of socio-economic and maternal human capital in this cycle is therefore crucial. Moreover, the longer-term consequences of low human capital for child health in South Asia, where early women's marriage and low education are widespread, have received little attention.

### Framework of analysis

**Figure 2:**
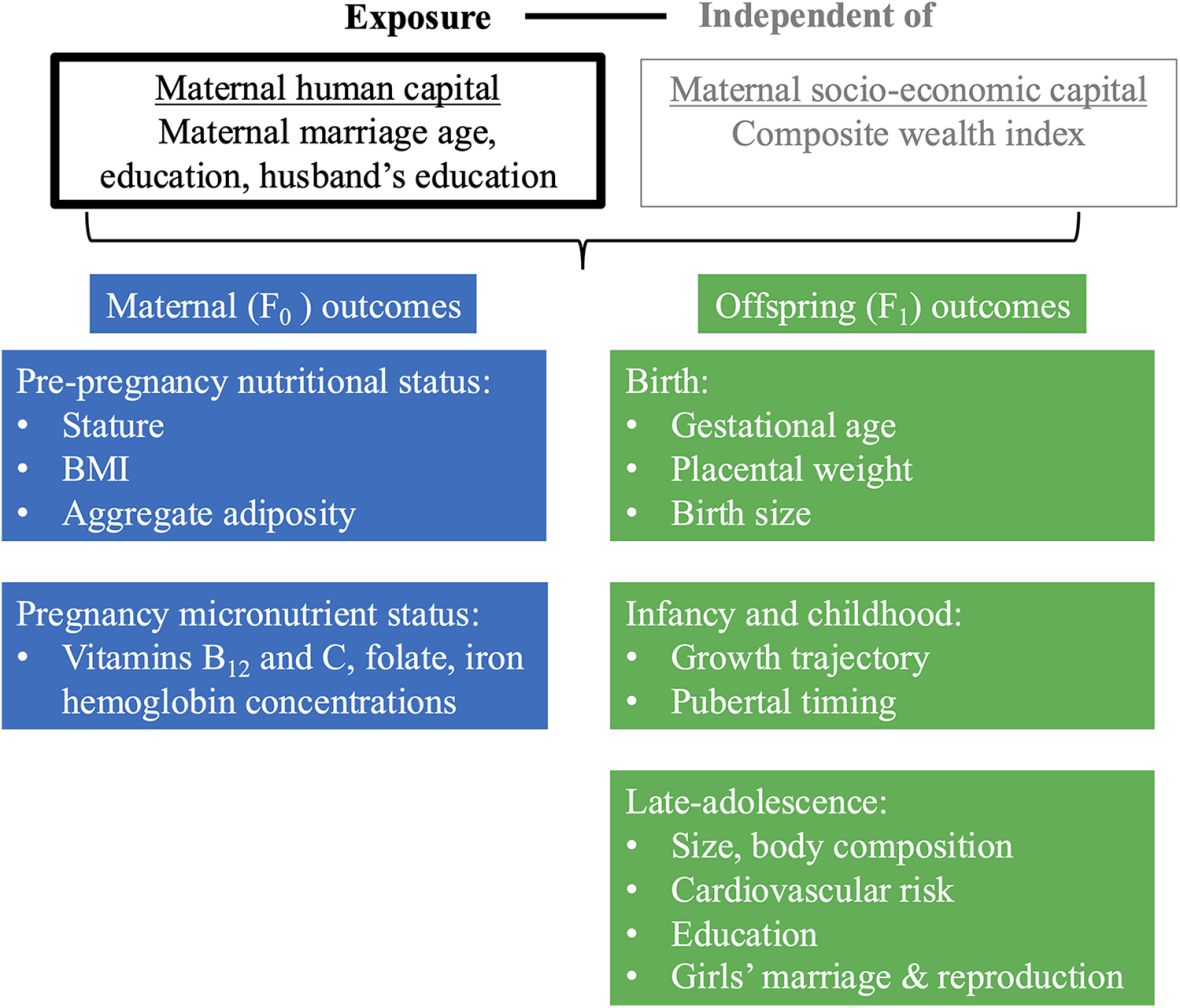
Framework of analysis. This schematic diagram of our framework of analysis illustrates our main exposure (in black text, in box outlined in black), ‘maternal human capital’, our secondary exposure, ’socio-economic capital’ for which we adjust (in grey text, in box outlined in grey), and our outcomes for the F_0_ mother at pre-pregnancy and pregnancy (blue boxes) and F_1_ offspring at birth, infancy/childhood and late-adolescence (green boxes). F_0_ refers to the maternal generation at baseline, and F_1_ to her offspring, or first generation. Regression analyses investigated the association of low and mid maternal human capital, relative to high human capital, independent of socio-economic capital, with maternal and offspring outcomes.

The framework of analysis in Figure 2 illustrates our approach, which draws on the different aspects of maternal capital illustrated in Figure 1. We aimed to evaluate whether maternal human capital is associated with a range of maternal and child outcomes, independent of socio-economic capital.

Our paper aims to break new ground in four areas. First, we focus on South Asia, where early marriage, early school drop-out and maternal and child undernutrition have been common (60, 63, 93, 101). Second, we take as our exposure women's human capital (of which maternal marriage age and education are key components) and analyse whether this is independent of socio-economic capital (of which wealth, caste affiliation and other aspects of socio-economic status are key aspects), thereby enabling us to identify which component of women's capital is relevant for improving maternal and offspring outcomes. Third, our markers of maternal human capital were measured prospectively before pregnancy, allowing us to robustly assess their association with maternal and child outcomes during pregnancy and at birth respectively. Fourth, due to the extended follow-up of the offspring in this cohort, we include in our analysis a wide range of nutritional, health and human capital outcomes up to the age of 24 years. We included cardiometabolic risk at young adulthood among these outcomes, as there is substantial evidence that variability in this component of health is both a consequence of early-life development, and may have implications for health of the following generation (56, 102–106). Overall, our approach enables us to examine whether an intergenerational cycle of disadvantage is being perpetuated by low human and/or socio-economic capital.

### Hypothesis

We use data on 618 mother-child dyads from the prospective Pune Maternal Nutrition Study in rural India. These dyads provide data from 2 generations: the maternal generation, which we refer to as baseline, or F_0_ generation, and their offspring, which we refer to as the first, or F_1_ generation. We test the following overarching hypothesis: that low human capital in the F_0_ mother is associated with F_0_ undernutrition before and during conception, and with poor growth, school drop-out, early marriage, early reproduction and greater cardiometabolic risk in F_1_ offspring.

## Materials and methods

### Study profile

We used data from the Pune Maternal Nutrition Study (PMNS), a prospective longitudinal birth cohort in rural Maharashtra state, India (9, 11, 107, 108). The study aimed to understand how maternal nutrition influences fetal growth and children's susceptibility to diabetes and cardiovascular disease (11). The study was conducted across six drought-prone villages, located 40–50 km from Pune city, spanning a population of approximately 35,000 (109). Most families had small landholdings and earned their livelihood through subsistence farming (110). In addition to fulfilling household unpaid care work (cooking, cleaning, childcare, etc), most (75%) of the mothers in our study worked as labourers or on farms (110). The majority of families were vegetarian (9). About 20% were nuclear families, 72% of households had electricity, 99% owned their homes and 82% owned land (agricultural and irrigated land) (111). Women generally have low autonomy and decision-making in households and tend to eat last despite working long hours on domestic tasks and farming (110, 112).

In Maharashtra state, at the time of our study, 44% of females (aged 6 + years) were illiterate, and 54% of women aged 20–24 years had married before 18 years of age (113). The society is traditionally patriarchal, with inheritance being passed down through the male line, and girls ending their schooling at marriage and moving to their husband's family to live with their in-laws (114). In India, caste affiliation reflects a person's status within a hierarchical social structure and is generally considered as a proxy for wealth and their overall socio-economic status (115, 116). A person's status within this social stratification generates inequalities, with those at the lowest ranks (e.g., scheduled castes and scheduled tribes) tending to have lower wealth and poorer health outcomes (115, 117).

**Figure 3:**
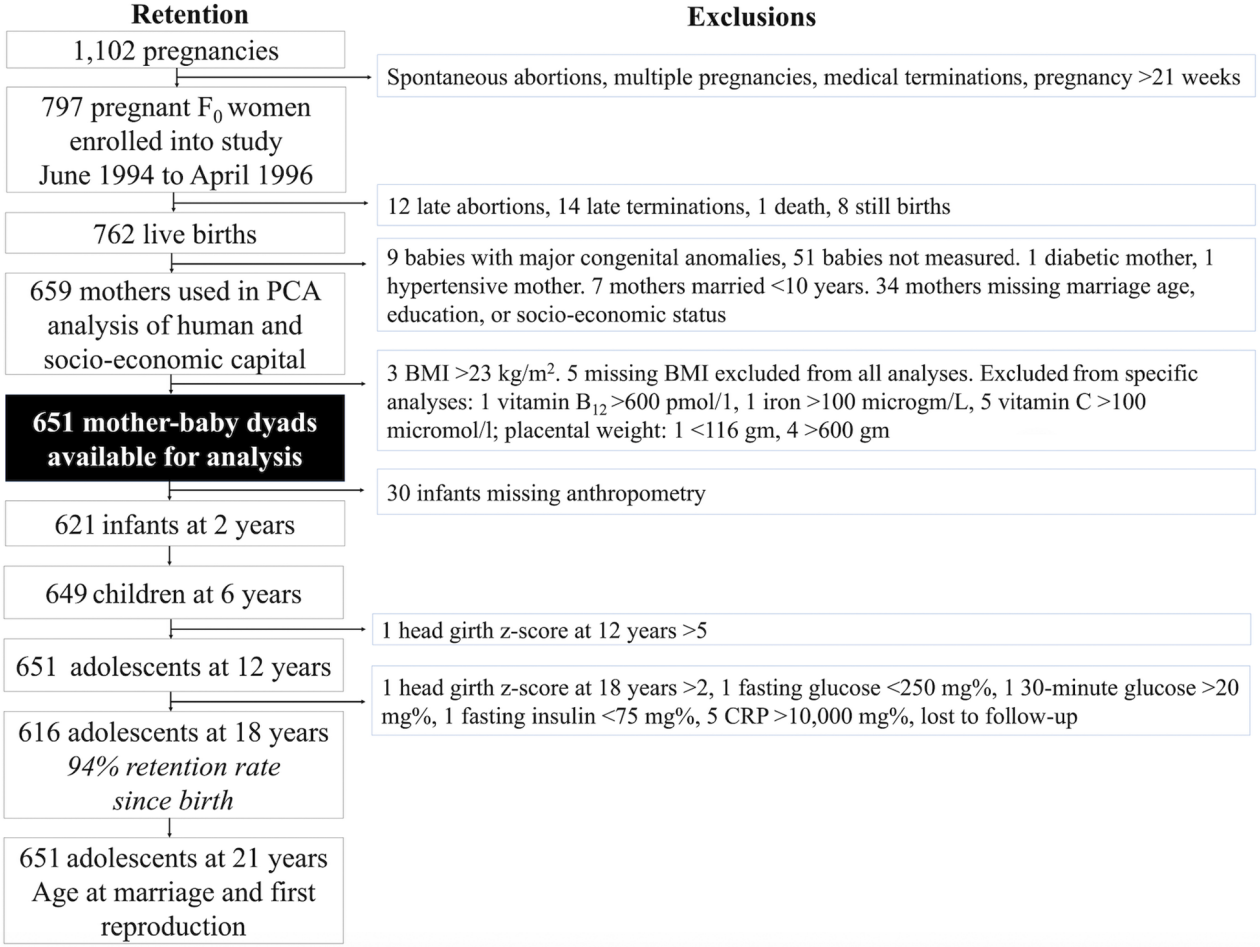
Pune Maternal Nutrition Study profile. This flow chart illustrates the study profile of the Pune Maternal Nutritional Study, including the number of women and offspring available for different analyses at different time-points, and the exclusions. Exclusions for exposure, outcome and control variables applied to all analyses whilst the other exclusions pertained only to analyses which used these data. F_0_ refers to the maternal generation at baseline, and F_1_ to her offspring, or first generation.

The flowchart in Figure 3 illustrates the PMNS profile and our sample selection at different time-points (118). Briefly, between 1993 and 1996, the PMNS identified 1,102 married, pregnant women of childbearing age residing in six villages of rural Pune district. Of these women, 797 with a singleton pregnancy of <21 weeks gestation were enrolled into the study between June 1994 and April 1996. Data collection started for all of the 797 women whose pregnancy was still <21 weeks at time of contact, and who had not had a spontaneous abortion, foetal anomalies, or medical terminations, and who were not carrying twins or triplets.

Further exclusions included mothers who had diabetes or hypertension, those who had married very early (<10 years), those missing data on key exposure variables (maternal marriage age, spousal education, socio-economic status), those who had babies with major congenital anomalies, and babies who were not measured at birth. These exclusions yielded 659 women for our Principal Component Analysis (PCA) of human and socio-economic capital. We arrived at a final sample of 651 mother-child dyads after excluding an additional 8 women for high or missing BMI. The sample size for maternal micronutrient analyses during pregnancy varied between 442 and 571 due to missing variables (Supplementary Table S1). Further exclusions for very low or high values in specific variables were taken.

Of the 651 mother-child dyads in our analysis, 30 were missing infant anthropometry, resulting in 621 infants at the age of 2 years. However, some of the offspring with missing birth or infant data re-entered the study at later time-points. At the age of 6 years, 649 offspring were followed-up, and at 12 years, 651. At the age of 18 years, 616 offspring were followed-up, representing a 94% retention rate from the 651 recruited at birth. When offspring were 24 years of age, data on age at marriage and (first) reproduction data were collected from all 651 participants recruited at birth by phone.

Ethical permission for the PMNS was granted by the local village leaders, and the Ethical Committee at the King Edward Memorial Hospital Research Centre (VSP/Dir.Off/EC/2166). Approval for the collection of data on the educational and marriage trajectories of offspring at the 18-years follow-up was provided by the King Edward Memorial Hospital Research Centre and the University of Cambridge Research Ethics Committee in the Department of Geography. The collaboration was approved by the Indian Health Ministry Screening Committee. As adolescents younger than the majority age of 18 years were not legally empowered to provide consent, their parents/guardians gave written consent for their participation in the study and adolescents themselves also provided written assent (or agreement). Upon turning 18 years of age, these adolescents then gave their own written consent to participate in the study.

## Measurements

### Maternal and household characteristics

Upon recruitment into the study, an oral questionnaire administered to married non-pregnant women (F_0_) recorded their age (years), age at marriage (years), parity (live births) and educational attainment (years of schooling completed). Husband's (F_0_) educational attainment (years), marital household socio-economic status (score), agricultural landholding (acres), family type (nuclear, joint) and caste affiliation (low: tribal, scheduled; mid: artisan, agrarian; or high: prestige, dominant) were also recorded at baseline. Caste affiliation was categorised according to the standardized Pareek and Trivedi “Socio-economic Status Scale” for rural India (119, 120). Household socio-economic status was measured using the widely used standardised Pareek and Trivedi socio-economic status scale for rural India (119, 120). The nine characteristics used to create this scale included: caste affiliation, occupation and education of the household head, land ownership, social participation, house infrastructure, farm power (number of draught animals), material possessions (bullock cart, cycle, radio, chairs, television, refrigerators), and family type (119, 120). Socio-economic status was assessed based on the characteristics of the individual and weighted score of each item (119, 120).

Women's (F_0_) pre-pregnancy anthropometry included height, measured to 0.1 cm (Harpenden portable stadiometer, CMS), weight to 0.5 kg (SECA digital scales, CMS), and subcutaneous skinfolds (biceps, triceps, subscapular and suprailiac), measured to 0.1 mm (Harpenden callipers, CMS) (121). Women's (F_0_) micronutrient status, measured at 28-weeks gestation, included vitamin B_12_ (pmol/L), haemoglobin (gm/dl), red cell folate (ng/ml), ferritin (microgm/L) and serum vitamin C (micromol/L), and has been described elsewhere (9, 122). Maternal smoking and alcohol use were also measured at 17-weeks gestation. Women (F_0_) were asked to answer ‘yes or no’ to whether they drank alcohol, smoked, chewed tobacco or betel leaf, and whether there were regular smokers in the house.

### Offspring characteristics

We used gestational age and placental weight as markers of offspring (F_1_) outcomes. Duration of pregnancy (weeks) was measured from the mother's (F_0_) last menstrual period, but if it differed from the sonographic estimate by more than two weeks, the latter estimate was used (9). Information on the sex of the foetus (F_1_) was not available to the families before birth. Placental weight was measured using the modified parallel planimetric method (123). Within 72 h of birth, offspring (F_1_) weight was measured to 25 g (spring balance, Salter Abbey) and crow-heel length to 0.1 cm (Pedobaby Babymeter ETS J.M.B). Duration of exclusive breastfeeding (months) was recorded by a questionnaire addressed to mothers. Children's (F_1_) standing height was measured to 0.1 cm at 2 years of age using a Harpenden stadiometer and at 6, 12 and 18 years by a wall-mounted microtoise (CMS). Weight was measured by electronic scales to 0.1 kg (ATCO Healthcare). Pubertal development was assessed by Tanner staging using clinical assessment. Girls’ age at menarche was also obtained.

When the offspring (F_1_) turned 18 years of age, data on smoking status, alcohol use, body composition [fat mass (kg) and fat free mass (kg)] and cardiovascular risk were recorded. Markers of cardiovascular risk included systolic and diastolic blood pressure (mmHg), three measurements of glucose (mg%) and insulin (mu/L): fasting, 30-minute and 2-hour, cholesterol (mg%), high-density lipoprotein (HDL, mg%), triglycerides (mg%), creatinine (mg%) and c-reactive protein (mg/dl). The primary outcome of the glucose tolerance test was the glucose value at 2-hour, used to categorise individuals as having normal glucose tolerance, pre-diabetes or diabetes. However, elevated glucose levels at 30 min also predict the development of diabetes in the longer term, as do low insulin at 30-minute and 2-hour (124). As the cohort were relatively young, the four outcomes were all reported to assess both current diabetes and future diabetes risk.

Data on offspring (F_1_) education, marriage and first reproduction were collected prospectively, at 18 and 24 years of age.

### Data coding

#### Exposures

Investigating the associations of age at marriage, education, and socio-economic status may be challenging because they are often highly correlated. Therefore, drawing on our previous analyses in Brazil (56, 102) and Nepal (125), we used PCA to examine how these individual variables may cluster around each other. We used continuous values of maternal marriage age, maternal and husband's education and household socio-economic status to derive two orthogonal composite scores of capital. We did not undertake the PCA with the aim of producing an index of human capital and an index of socio-economic capital, but rather to see whether there were different components of capital, and what they represented.

The two components that emerged from the PCA captured two different axes of variability (Biplot shown in Supplementary Figure S1), and were best characterised as human and socio-economic capital. The very weak correlation of the two PC scores of 0.09 indicates that variability in one explains only 1% of the variability in the other, ie they are essentially independent.

The first component score derived from PCA explained 44% of variance. The variables contributing the highest factor loading included: maternal education (0.884), husband's education (0.817), and maternal marriage age (0.536). We interpret this component as ‘maternal human capital’. We interpret the second PCA component as ’socio-economic capital’. This component explained 24% of variance and was primarily determined by socio-economic status (0.996), which as explained earlier, indexed household material possession and assets, land holding and other characteristics.

Although disentangling socio-economic and human capital can be challenging as proxy indicators such as education can plausibly contain elements of both, our PCA clearly indicated that education coalesced around marriage age rather than socio-economic status. Hence, the education components that were captured by the ‘human capital’ PCA were not reflected in the ’socio-economic capital’ PCA.

The continuous scores derived from the 2 PCA components were then coded into tertiles, indicating low to high (1–3) levels of maternal human and socio-economic capital respectively. We categorised the PCA components to evaluate whether a particular threshold, indicated by the categorisation low, mid or high, was associated with our maternal and offspring outcomes. This approach enables us to identify which of maternal human capital or socio-economic capital is the most relevant factor for maternal and offspring outcomes.

#### Outcomes

We used outcomes relating to maternal (F_0_) physiological traits and micronutrient status as continuous values. We derived pre-pregnancy body mass index (BMI) from weight and height (kg/m^2^). We constructed an aggregate (F_0_) pre-pregnancy adiposity index by averaging five standard deviation scores (z-scores), generated internally for BMI, and four skinfolds (biceps, triceps, subscapular, and suprailiac).

Outcomes relating to offspring (F_1_) phenotype were coded as continuous values. We calculated age- and sex-specific anthropometric z-scores for height (cm), weight (kg), and BMI (kg/cm^2^) at birth, 2-, 6-, 12-, and 18-years using UK 1990 rather than WHO anthropometric reference data. We used the UK reference data because it adjusts for gestational age and provides a single reference throughout children's development, including puberty (126, 127). The conversion of individual data-points to z-scores (SDS) requires reference values for three variables, namely M (mu, or the median), S (sigma, or the coefficient of variation) and L (lambda, the Box-Cox power needed to normalise the data). However, the UK 1990 reference data only provide LMS values for head circumference up to 18 years for boys and 17 years for girls. As the age distribution of the 18-year PMNS follow-up fell only just beyond these values, we predicted M and S values for each sex, by generating quadratic equations for the association of M and S with age in the UK 1990 dataset and applying these equations to subsequent ages. There was no need to do this for L values, as these had a universal value of 1 for all ages in both sexes. All quadratic equations had R^2^ of 1, indicating excellent fit.

Binary outcomes for offspring (F_1_) were created for preterm birth (<37 weeks) (128), low birthweight (<2.5 kg) and low exclusive breastfeeding (<6 months). Not completing secondary school indicated not completing the 12th standard (99). In a sub-sample of adolescents (F_1_) with available data, we explored factors associated with having pre-diabetes, which included the one girl who had diabetes at 18 years. Our analysis of (F_1_) early marriage (<19 years) and first reproduction (<20 years) was restricted to girls as only one boy was married by 19 years, and none had reproduced by 20 years. As in a previous study (98), we used <19 years as the cut-off for early marriage as a greater proportion of girls in our cohort were married by this age compared to the UN minimum marriage age threshold of 18 years. Other studies have also found that girls marrying just before and after 18 years have similar experiences (129, 130). We also describe the median age at marriage and first reproduction for girls (F_1_) at 24 years of age, but do not use them in regression models as our interest was in understanding lower values of these outcomes, early marriage and early first reproduction.

Data on smoking and alcohol use were collected at both maternal (F_0_) pre-pregnancy and the adolescent (F_1_) 18-year follow-up. Although participants responded to this question, none of them smoked or consumed alcohol. These data were therefore not used in our analyses.

We assessed secular trends between mothers (F_0_) and daughters (F_1_) for height, education and marriage age. We reported mean heights and median years of education, and age at marriage, and calculated the difference between mothers and daughters, but did not apply statistical tests. As not all daughters (F_1_) had completed their education or got married, we ranked all daughters in terms of their years of schooling completed and marriage age and took as the median the value of the relevant middle-ranked daughter.

### Statistical analysis

We report the sample size for each variable in each analysis because it differed slightly across variables measured at different time-points. Normally distributed continuous data on mothers and offspring were described using mean and standard deviation (SD). Categorical variables were described as frequencies and percentages. We summarised women's age (F_0_ at pre-pregnancy) and age at marriage (for both F_0_ and F_1_) with median, and the 25th percentile [Quartile 1 (Q1)] and 75th percentile [Quartile 3 (Q3)], and log-transformed fat mass and C-reactive protein at (F_1_) 18 years, given the skewed distribution of these data. For continuous variables, we used Independent Samples *t*-test to assess differences in normally distributed variables and the Kruskal-Wallis Test for non-normally distributed variables. Chi-square tests were used to assess differences in categorical variables.

Ordinary Least Squared (OLS) linear regression investigated the change in units (ß with 95% Confidence Interval, CI) in outcome variables associated with low and mid maternal (F_1_) human capital, relative to high human capital, and independent of (F_1_) socio-economic capital. Whilst our primary focus is on maternal human capital, we also reported associations of socio-economic capital as this allowed us to evaluate the relative importance of maternal human capital. Multivariable logistic regression investigated the odds (adjusted Odds Ratio, aOR with 95% CI) of offspring (F_1_) being in a risk group associated with low and mid maternal (F_0_) human capital, independent of F_0_ socio-economic capital. To interpret aORs <1, we subtracted the aOR from 1 and multiplied by 100, which yields the reduced risk in percentage terms. We do not adjust for multiple comparisons as our interest was in understanding the magnitude of the effect (ß or aOR with 95% CI). In the statistical tables, we report the *p*-values for information only, but do not evaluate them as a finding.

Dummy variables for a variety of categorical variables were created for linear regression models. The reference groups were the highest maternal (F_0_) human and socio-economic capital groups and males [(F_1_) in offspring models]. Models controlled for maternal (F_0_) age (continuous value, in years) and parity (0, 1 and ≥ live births), with parity 0 set as the reference group.

Analyses were conducted in SPSS version 27 (131).

## Results

Supplementary Table S1 lists missing data on maternal and offspring traits and tests for bias by maternal F_0_ human capital, F_0_ socio-economic capital, maternal age, maternal stature and BMI. Overall, very few women were missing data on exposures. Younger F_0_ women (aged <20 years) were more likely to be missing data on their marriage age, on their own and their husband's education, and their haemoglobin and iron. Low human capital mothers were also more likely to have missing data on their vitamin B_12_ and haemoglobin. Low human and socio-economic capital mothers had more missing data on their serum vitamin C. Offspring (F_1_) with missing anthropometric data at 2 years were more likely to have higher BMI mothers. F_1_ missing anthropometric, schooling and biomarker data at 18 years were more likely to come from lower maternal human capital households and higher BMI mothers. However, the magnitude of these differences was minimal and therefore not expected to bias our results.

### Description of sample

The biplot shown in Supplementary Figure S1 shows our exposure variables, derived by PCA. ‘Maternal human capital’ represented maternal marriage age, maternal and husband's education (132). ‘Socio-economic capital’ represented household wealth and other socio-economic status variables (score). Supplementary Table S2 describes each of the traits included in these composite components of capital. The low and mid maternal human capital groups had similar median marriage ages. The low human capital group had lower median values of maternal and husband's education than the mid human capital group.

### Maternal (F_0_) outcomes

Table 1 describes maternal and household characteristics for the 651 women included in our analysis. At recruitment into PMNS, mothers were of median age of 21 years (Q1: 19, Q3: 23), and their median age at marriage was 18 years (Q1: 16, Q3: 19). Thirty-one percent of mothers were primiparous, 34% had one previous birth and 35% had 2 or more previous births. Mothers were on average 152 cm tall and had low BMI (18 kg/m^2^). Mothers and their husbands had low educational attainment, having completed just under six and eight years of schooling respectively. Nine percent of households were from low caste groups, 23% from mid and 68% from high caste groups.

**Table 1:** Description of F_0_ maternal and F_0_ household characteristics (*n* = 651).

Maternal characteristics, measured at pre-pregnancy		
	*n*	Median (Q1, Q3)
Maternal age (years)	651	21 (19, 23)
Maternal age at marriage (years)	651	18 (16, 19)
	*n*	Mean (SD)
Maternal education (years)	651	5.7 (3.9)
Height (cm)	651	152.0 (4.9)
BMI (kg/m^2^)	651	18.0 (1.8)
Aggregate adiposity (SDS)	651	0.03 (0.8)
	*n*	F (%)
Parity (no. of live births)	651	
0 previous births		200 (31)
1 birth		224 (34)
≥2 births		227 (35)
Household characteristics, measured at maternal pre-pregnancy		
	*n*	Mean (SD)
Husband's education (years)	651	7.8 (3.9)
Socio-economic status (score)	651	26.9 (6.6)
	*n*	F (%)
Caste affiliation	648	
Low (tribal, scheduled)		59 (9)
Mid (artisan, agrarian)		149 (23)
High (prestige, dominant)		440 (68)
Maternal pregnancy, measured at maternal 28-weeks gestation		
	*n*	Mean (SD)
Vitamin B_12_ (pmol/L)	552	140 (75)
Haemoglobin (gm/dl)	571	11.1 (1.5)
Red cell folate (ng/ml)	517	458 (179)
Iron (microgm/L)	547	15.0 (11.6)
Serum Vitamin C (micromol/L)	442	16.6 (18.8)

F_0,_ maternal generation; *n,* number; Q1, 25th percentile; Q3, 75th percentile; SD, Standard Deviation; *F*, frequency; %, percentage; SDS, *z*-score.

Table 2 shows linear regression models associating maternal human capital, independent of socio-economic capital, with maternal outcomes. We focus here on the magnitude of the effect and 95% CI of low maternal human capital but note that results were broadly similar for the mid maternal human capital group. We report *p*-values in tables for information. Compared to high maternal human capital, low human capital was associated with shorter maternal stature −1.0 cm (95% CI −1.9, −0.1), lower adiposity −0.2 SDS (95% CI −0.3, −0.1), higher vitamin B_12_ 17.2 pmol/L (95% CI 1.3, 33.1) and lower red cell folate ng/ml −64.9 (95% CI −103, −26.8), independent of socio-economic capital. Compared to women of high human capital, there were no associations between maternal thinness, haemoglobin, iron or vitamin C and mid human or socio-economic capital.

**Table 2:** OLS linear regression of F_0_ maternal human capital and F_0_ maternal outcomes.

	F_0_ exposures								
	PCA 1: Maternal human capital (ref: high capital)				PCA 2: Socio-economic capital (ref: high capital)				
	Low capital		Mid capital		Low capital		Mid capital		
F_0_ maternal outcomes	ß (95% CI)	*p*-value	ß (95% CI)	*p*-value	ß (95% CI)	*p*-value	ß (95% CI)	*p*-value	*n*
Height (cm)	−1.0 (−1.9, −0.1)	0.035	−0.8 (−1.7, 0.2)	0.102	−1.2 (−2.1, −0.3)	0.010	−0.8 (−1.8, 0.1)	0.072	651
BMI (kg/m^2^)	−0.1 (−0.5, 0.2)	0.579	−0.1 (−0.4, 0.3)	0.606	0.1 (−0.4, 0.3)	0.723	−0.1 (−0.5, 0.2)	0.447	651
Aggregate adiposity (SDS)	−0.2 (−0.3,−0.1)	0.017	−0.1 (−0.3, 0.1)	0.071	0.1 (−0.1, 0.3)	0.134	0.1 (−0.2, 0.2)	0.951	651
Vitamin B_12_ (pmol/L)	17.2 (1.3, 33.1)	0.034	13.2 (−2.5, 28.8)	0.100	−6.9 (−22.3, 8.6)	0.384	−6.5 (−21.7, 8.8)	0.407	552
Haemoglobin (gm/dl)	−0.1 (−0.3, 0.3)	0.899	−0.2 (−0.5, 0.1)	0.177	−0.1 (−0.4, 0.3)	0.760	−0.2 (−0.6, 0.1)	0.074	571
Red cell folate (ng/ml)	−64.9 (−103, −26.8)	0.000	−65.7 (−104, −27.8)	0.000	0.1 (−37.3, 37.5)	0.996	−10.3 (−47.0, 26.4)	0.580	517
Iron (microgm/L)	−1.5 (−3.9, 0.9)	0.224	−2.5 (−4.7, −0.1)	0.065	−0.4 (−2.7, 2.0)	0.766	−2.2 (−4.5, 0.1)	0.059	547
Vitamin C (micromol/L)	−1.8 (−6.2, 2.7)	0.437	−0.1 (−4.5, 4.2)	0.951	−2.4 (−6.8, 2.0)	0.292	−0.1 (−4.2, 4.1)	0.988	442

F_0,_ maternal generation; CI, confidence interval; *n*, number; SDS, *z*-score. Models control for maternal age (continuous value, years) and parity (ref = 0).

### Offspring (F_1_) outcomes

Table 3 describes offspring phenotype and assesses differences between girls and boys. The average duration of gestation was 39 weeks and exclusive breastfeeding eight months; 31% of infants were exclusively breastfed for <6 months. About 10% of infants were born preterm and 30% were of low birthweight (<2.5 kg). Supplementary Table S3 shows differences by offspring sex in size and nutritional status (z-scores) from birth to 18 years and pubertal age. Girls were smaller at birth than boys, had lower head circumference z-score at 6 and 12 years and lower BMI z-score at 12 years. By 18 years, other than lower weight and BMI z-scores, differences in stature and head circumference had attenuated, indicating some catch-up during adolescence.

**Table 3:** Description of F_1_ offspring phenotype, from birth to 24 years of age, stratified by sex.

	Full sample		Girls		Boys		Difference^a^	
	*n*	Mean (SD)	*n*	Mean (SD)	*n*	Mean (SD)	Δ (95% CI)	*p*-value
F_1_ Offspring traits as continuous outcomes (for OLS Linear Regression)								
Gestation (weeks)	651	39.0 (1.7)	310	39.1 (1.6)	341	39.0 (1.8)	0.1 (−0.2, 0.4)	0.454
Placental weight (gm)	565	359.2 (71.0)	264	352.4 (71.5)	301	365.2 (70.2)	−12.8 (−24.5, −1.1)	0.032
Exclusive breast feeding (months)	647	8.3 (5.8)	308	8.0 (5.2)	339	8.5 (6.2)	−0.4 (−1.3, 0.4)	0.324
F_1_ Offspring traits as binary outcomes (for Logistic Regression)								
	*n*	F (%)	*n*	F (%)	*n*	F (%)	*p*-value^b^	
Preterm birth (<37 weeks)	651	64 (10)	310	31 (10)	341	33 (10)	0.890	
Low birthweight (<2.5 kg)	617	187 (30)	293	112 (38)	324	75 (23)	0.000	
Lower exclusive breastfeeding (<6 months)	647	198 (31)	308	85 (28)	339	113 (33)	0.114	
School drop-out (<12th standard)	611	121 (20)	286	66 (23)	325	55 (17)	0.057	
Pre-diabetes at 18 years (yes)	426	116 (27)	196	35 (18)	230	81 (35)	0.000	
Early marriage (<19 years)	651	73 (11)	310	72 (23)	341	1 (0.3)	na	
Early reproduction (<20 years)	212	52 (25)	189	52 (28)	23	0 (0)	na	
F_1_ Offspring marriage and reproduction at 24 years of age								
	*n*	F (%)	*n*	F (%)	*n*	F (%)	*p*-value^b^	
Married (yes)	651	339 (52)	310	246 (79)	341	93 (27)	0.000	
Reproduced (yes)	651	212 (33)	310	189 (61)	341	23 (7)	0.000	

F_1_, offspring generation; *n,* number; SD, standard deviation; *F*, frequency; %, percentage; ∆, Difference girls minus boys; CI, confidence interval; na, not applicable. ^a^Independent Samples *t*-test. ^b^Chi-squared test.

**Figure 4:**
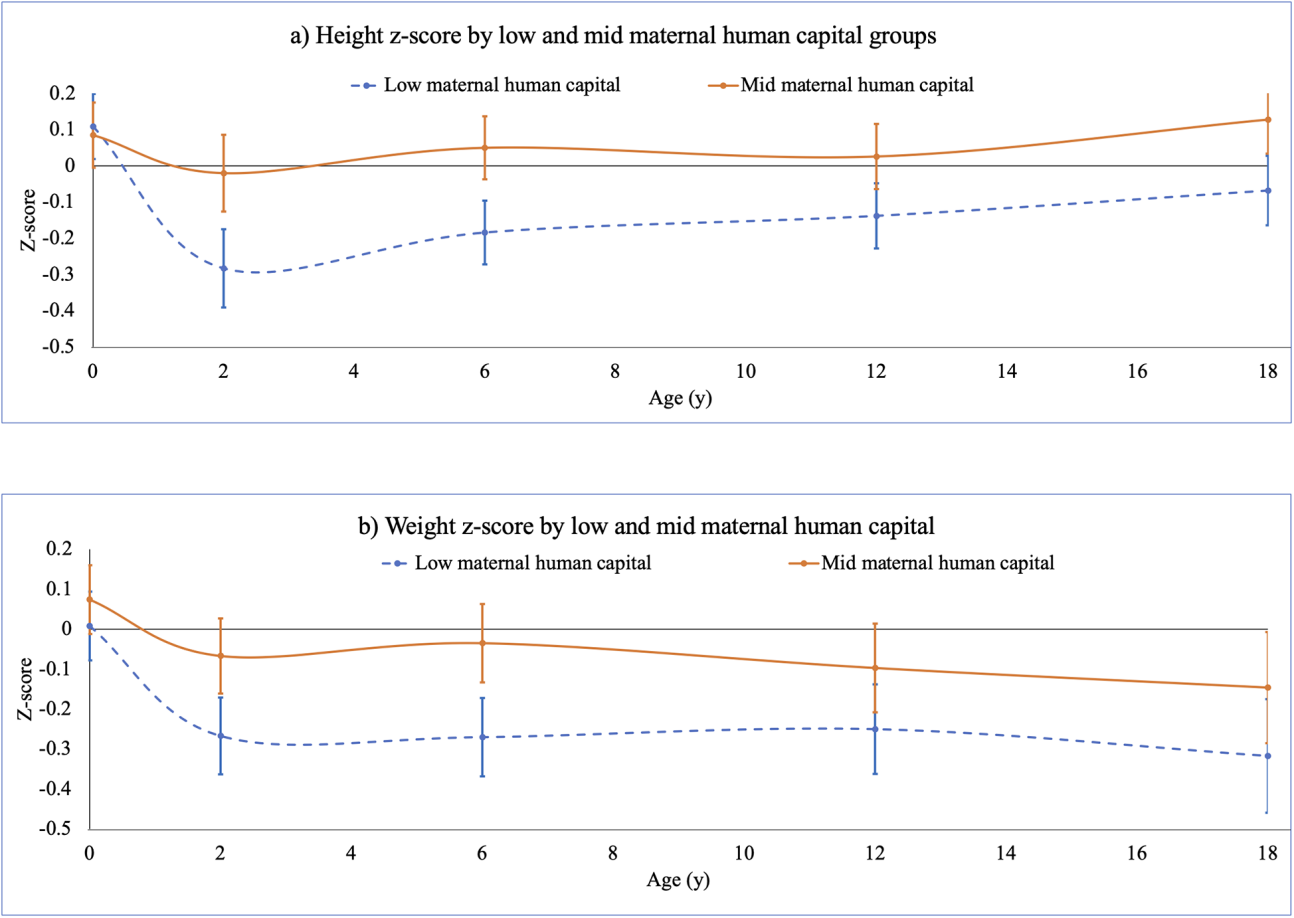
F_1_ offspring **(a)** height and **(b)** weight *z*-scores, birth to 18 years, stratified by low and mid maternal human capital. This figure illustrates results of linear regression models associating F_0_ low and mid maternal human capital groups, relative to high maternal human capital (zero line), with F_1_ offspring growth trajectory from birth to 18 years (data in Supplementary Table S4). Models controlled for F_0_ socio-economic capital, and adjusted for F_0_ maternal age, parity. F_1_ age- and sex-specific height and weight z-scores were computed using UK 1990 (Least Mean Squares (LMS) option in Microsoft Excel™ (133). Low F_0_ maternal human capital was associated with lower F_1_ height and weight z-scores at 2, 6 and 12 years, and lower weight z-score at 18 years.

Figure 4 shows that adjusting for these sex differences, and for socio-economic capital, low maternal human capital was associated with lower height z-score and weight z-scores at 2, 6 and 12 years and weight z-score at 18 years. Supplementary Table S4 shows these results and associations of low maternal human capital with lower head circumference z-score at 2 years −0.2 (95% CI −0.4, −0.1) and lower BMI *z*-score at 6 years −0.2 (95% CI −0.4, −0.1) and at 18 years −0.3 (95% CI −0.6, −0.1). Further analysis showed that differences in height *z*-score at 2 years associated with low maternal human capital persisted even after adjusting for maternal height.

Table 3 also shows that 20% of adolescents had left school before obtaining their secondary school certificate (awarded upon completing the 12th standard). By the age of 24 years, 52% of adolescents were married (79% of girls and 27% of boys) and 33% had reproduced (61% of girls and 7% of boys). Compared to boys (17%), 23% of girls had left secondary school. About 35% of boys and 18% of girls had pre-diabetes at the age of 18 years. Only one boy married early (<19 years) compared to 23% of girls and none of the boys had reproduced early, and 28% of girls had reproduced before 20 years of age. The median age at marriage of girls was 20.7 years (Q1: 19.2, Q3: 25.9). The median age of first reproduction of girls was 21.4 years (Q1: 20.0, Q3: 22.9).

Table 4 shows that independent of socio-economic capital, low maternal (F_0_) human capital was associated with lower gestation −0.4 weeks (95% CI −0.7, −0.1), lower F_1_ birth head girth −0.3 cm (95% CI −0.6, −0.1) but longer exclusive breastfeeding of 2.6 months (95% CI 1.5, 3.7). There were no associations between maternal human capital and placental weight, birthweight, birth length, F_1_ girls’ age at menarche or pubertal timing. Separate regression models by offspring sex of pubertal timing confirmed the null association of maternal human capital. Independent of maternal human and socio-economic capital, girls differed from boys at birth by having a lower placental weight −12.1 gm (95%CI −23.9, −0.4), lower weight −0.1 kg (95% CI −0.2, −0.1), being shorter −0.7 cm (95% CI −0.1, −0.3) and having a smaller head girth −0.6 cm (95% CI −0.8, −0.3).

**Table 4:** OLS linear regression of F_0_ maternal human capital and F_1_ outcomes.

	F_0_ exposures								F_1_ outcome		
	PCA 1: Maternal human capital (ref: high capital)				PCA 2: Socio-economic capital (ref: high capital)				Offspring sex (ref: boys)		
	Low capital		Mid capital		Low capital		Mid capital		Girl		
F_1_ outcomes	ß (95% CI)	*p*-value	ß (95% CI)	*p*-value	ß (95% CI)	*p*-value	ß (95% CI)	*p*-value	ß (95% CI)	*p*-value	*n*
Gestation (weeks)	−0.4 (−0.7, −0.1)	0.023	−0.3 (−0.6, 0.1)	0.089	−0.1 (−0.4, 0.4)	0.741	0.4 (0.1, 0.7)	0.839	0.1 (−0.2, 0.4)	0.367	651
Placental weight (gm)	−13.6 (−28.3, −1.1)	0.070	−12.6 (−27.2, 1.9)	0.088	−10.7 (−25.0, 3.6)	0.143	−9.7 (−23.8, 4.4)	0.179	−12.1 (−23.9, −0.4)	0.043	565
Birthweight (kg)	−0.1 (−0.1, 0.1)	0.173	−0.1 (−0.1, 0.1)	0.654	−0.1 (−0.1, 0.1)	0.596	−0.1 (−0.1, 0.1)	0.036	−0.1 (−0.2, −0.1)	0.000	617
Birth length (cm)	−0.1 (−0.6, 0.3)	0.558	−0. 1 (−0.6, 0.3)	0.575	−0.3 (−0.8, 0.1)	0.131	−0.4 (−0.8, 0.1)	0.077	−0.7 (−1.0, −0.3)	0.000	641
Birth head girth (cm)	−0.3 (−0.6, −0.1)	0.018	−03 (−0.5, 0.1)	0.064	0.0 (−0.3, 0.3)	0.999	−0.2 (−0.4, 0.1)	0.241	−0.6 (−0.8, −0.3)	0.000	641
Duration of breastfeeding (months)	2.6 (1.5, 3.7)	0.000	0.7 (−0.4, 1.8)	0.230	0.5 (−0.6, 1.6)	0.360	−0.2 (−1.3, 0.9)	0.714	−0.6 (−1.5, 0.3)	0.168	647
Girls’ age at menarche (years)	0.1 (−0.2, 0.4)	0.602	0.1 (−02, 0.4)	0.525	−0.1 (−0.4, 0.3)	0.807	0.2 (−0.1, 0.5)	0.210	na	na	305
Pubertal timing (years)	−0.1 (−0.2, 0.2)	0.898	−0.1 (−0.3, 0.1)	0.134	0.1 (0.1, 0.4)	0.161	−0.1 (−0.2, 0.2)	0.891	0.1 (−0.1, 0.3)	0.128	617

F_0_, maternal generation; F_1_, offspring generation; CI, confidence interval; *n*, number; na, not applicable. Models control for maternal age (continuous value, years) and parity (ref = 0).

Supplementary Table S5 shows logistic regression models of offspring outcomes. We report associations of low maternal human capital but note that trends were broadly similar, though the magnitude of the effect was weaker in the mid human capital group. Independent of socio-economic capital, offspring of low maternal human capital mothers had a 60% (95% CI 0.2, 0.6) reduced likelihood of being exclusively breastfed for less than 6 months. Offspring of low maternal human capital groups were 4.2 times (95% CI 2.4, 7.4) more likely to drop out of secondary school. Girls-only models showed that those with low maternal human capital were 2.7 times (95% CI 1.3, 5.5) and 2.2 times (95% CI 0.9, 5.2) more likely to marry early and 2.2 times (95% CI 0.9, 5.2) to reproduce early. There were no associations between human capital and preterm birth, low birthweight and pre-diabetes status at 18 years.

Table 5 describes offspring cardiometabolic markers at 18 years. Girls differed from boys across several biomarkers: they had higher fat mass 9.4 kg (95% Q1 9.1, Q3 9.6), diastolic blood pressure 1.9 mmHg (95% CI 0.7, 3.1), cholesterol 8.2 mg% (95% CI 4.8, 11.8), HDL 3.8 mg% (95% CI 2.2, 5.3) and 2-hour insulin 16.2 mu/L (95% CI 7.0, 25.4). However, girls had lower fat free mass −14.2 kg (95% CI −15.0, −13.5), systolic blood pressure −7.6 mmHg (95% CI −9.0, −6.2), fasting glucose −4.6 mg% (95% CI −5.6, −3.7), triglycerides −8.0 mg% (95% CI −12.0, −4.0) and creatinine −0.2 mg% (95% CI −0.2, −0.1). There were no differences between girls and boys in 30-minutes and 2-hour glucose, fasting and 30-minute insulin. Taking these differences into account, linear regression models showed few associations between maternal human capital and cardiometabolic biomarkers at 18 years.

**Table 5:** Description of F_1_ biomarkers at 18 years.

F_1_ outcomes	Full sample		Girls		Boys		Difference	
	*n*	Median (Q1, Q3)	*n*	Median (Q1, Q3)	*n*	Median (Q1, Q3)	*p*-value^b^	
Fat mass (kg)^a^	611	9.2 (8.7, 9.6)	280	9.4 (9.1, 9.6)	331	8.9 (8.4, 9.3)	0.000	
C-reactive protein (mg/dl)^a^	610	−3.8 (−4.6, −2.7)	285	−3.9 (−4.8, −2.9)	325	−3.8 (−4.5, −2.6)	0.411	
	*n*	Mean (SD)	*n*	Mean (SD)	*n*	Mean (SD)	Δ (95% CI)	*p*-value^c^
Fat free mass (kg)	611	40.6 (8.6)	280	32.9 (3.6)	331	47.1 (5.7)	−14.2 (−15.0 −13.5)	0.000
Systolic blood pressure (mmHg)	617	109.8 (9.8)	285	105.7 (8.1)	332	113.3 (9.8)	−7.6 (−9.0, −6.2)	0.000
Diastolic blood pressure (mmHg)	617	60.2 (7.7)	285	61.2 (6.7)	332	59.3 (8.5)	1.9 (0.7, 3.1)	0.002
Fasting glucose (mg%)	616	95.1 (6.5)	284	92.6 (5.8)	332	97.3 (6.3)	−4.6 (−5.6, −3.7)	0.000
30-minute glucose (mg%)	582	149.3 (20.9)	253	148.9 (20.8)	329	149.6 (21.0)	−0.8 (−4.2, 2.7)	0.666
2-hour glucose (mg%)	580	112.8 (23.5)	252	114.2 (24.6)	328	111.8 (22.7)	2.3 (−1.5, 6.2)	0.234
Cholesterol (mg%)	617	129.0 (23.4)	285	133.4 (22.8)	332	125.2 (23.4)	8.2 (4.5, 11.8)	0.000
High-density lipoprotein (HDL) (mg%)	617	40.8 (10.2)	285	42.9 (9.0)	332	39.1 (10.7)	3.8 (2.2, 5.3)	0.000
Triglycerides (mg%)	617	61.8 (26.2)	285	57.5 (22.4)	332	65.5 (28.5)	−8.0 (−12.0, −4.0)	0.000
Creatinine (mg%)	617	0.7 (0.2)	285	0.6 (0.1)	332	0.8 (0.1)	−0.2 (−0.2, −0.1)	0.000
Fasting insulin (mu/L)	614	10.7 (5.1)	283	11.0 (4.2)	331	10.5 (5.8)	0.7 (−0.1, 1.5)	0.103
30-minute insulin (mu/L)	581	100.6 (64.8)	252	104.0 (51.8)	329	98.0 (73.2)	5.9 (−4.3, 16.1)	0.254
2-hour insulin (mu/L)	577	68.5 (56.8)	249	77.7 (53.9)	328	61.5 (58.1)	16.2 (7.0, 25.4)	0.000

F_1_, offspring generation; *n*, number; Q1, 25th percentile; Q3, 75th percentile; SD, standard deviation; Δ, Difference girls minus boys. CI, confidence interval.

^a^ log-transformed.

^b^ Kruskal-Wallis test.

^c^ Independent samples *t*-test.

Table 6 shows that independent of socio-economic capital, several F_1_ offspring biomarkers were associated with low and mid maternal human capital. Below, we describe the results for low human capital, but associations were similar for mid human capital, though the magnitude of the effect was smaller. Low maternal human capital was associated with lower fat mass −0.2 kg (95% CI −0.3, −0.1), 30-minute insulin −16.9 mu/L (95% CI −30.2, −3.6). Only mid maternal human capital was associated with lower triglycerides −6.2 mg% (95% CI −11.8, −1.2). Compared to boys, girls had higher fat mass 0.5 kg (95% CI 0.4, 0.6), diastolic blood pressure 2.1 mmHg (95% CI 0.8, 3.3), cholesterol 8.3 mg% (95% CI 4.5, 11.9), HDL 3.7 mg% (95% CI 2.1, 5.3) and 2-hour insulin 17.1 mu/L (95% CI 6.2, 25.4). However, girls had lower fat free mass −14.2 kg (95%CI −15.0, −13.4), systolic blood pressure −7.5 mmHg (95% CI −9.0, −6.0), fasting glucose −4.7 mg% (95% CI −5.6, −3.7), triglycerides −7.7 mg% (95%CI −11.8, −3.3) and creatinine −0.2 (95% CI −0.2, −0.1). We explored whether poor growth mediated the lower 30-minute insulin result at 18 years, but did not find any evidence of this.

**Table 6:** OLS linear regression of F_0_ maternal human capital and F_1_ biomarkers at 18 years of age.

	F_0_ exposures								F_1_ outcome		
	PCA 1: Maternal human capital (ref: high capital)				PCA 2: Socio-economic capital (ref: high capital)				Offspring sex (ref: boys)		
	Low capital		Mid capital		Low capital		Mid capital		Girl		
F_1_ outcomes	ß (95% CI)	*p*-value	ß (95% CI)	*p*-value	ß (95% CI)	*p*-value	ß (95% CI)	*p*-value	ß (95% CI)	*p*-value	*n*
Fat mass (kg)^a^	−0.2 (−0.3, −0.1)	0.000	−0.1 (−0.2, −0.1)	0.029	0.1 (−0.1, 0.2)	0.080	−0.1 (−0.1, 0.1)	0.753	0.5 (0.4, 0.6)	0.000	611
Fat free mass (kg)	−0.04 (−1.0, 0.9)	0.936	−0.3 (−0.6, 1.3)	0.495	−0.7 (−1.6, 0.3)	0.163	−1.0 (−1.9, −0.02)	0.047	−14.2 (−15.0, −13.4)	0.000	611
Systolic blood pressure (mmHg)	−0.9 (−2.8, 0.9)	0.317	−1.5 (−3.3, 0.3)	0.110	0.7 (−1.0, 2.5)	0.417	0.7 (−1.1, 2.4)	0.453	−7.5 (−9.0, −6.1)	0.000	617
Diastolic blood pressure (mmHg)	−1.4 (−2.9, 0.2)	0.085	−0.9 (−2.4, 0.6)	0.231	1.4 (−0.1, 2.9)	0.069	0.5 (−1.0, 2.0)	0.490	2.1 (0.8, 3.3)	0.000	617
Fasting glucose (mg%)	0.1 (−1.2, 1.2)	0.970	0.5 (−0.7, 1.7)	0.401	−0.2 (−1.4, 1.0)	0.747	0.1 (−1.0, 1.3)	0.804	−4.7 (−5.6, −3.7)	0.000	616
30-minute glucose (mg%)	2.4 (−2.1, 6.9)	0.280	−0.2 (−4.6, 4.2)	0.916	−2.7 (−9.8, 0.4)	0.247	−2.7 (−8.1, 1.0)	0.204	−1.0 (−5.2, 1.9)	0.586	582
2-hour glucose (mg%)	1.3 (−3.6, 6.1)	0.608	0.2 (−4.6, 5.0)	0.949	−0.9 (−5.6, 3.8)	0.715	−1.2 (−5.9, 3.5)	0.624	2.3 (−1.6, 6.2)	0.246	580
Cholesterol (mg%)	0.1 (−4.6, 4.7)	0.986	−1.4 (−6.0, 3.1)	0.539	0.3 (−4.3, 4.7)	0.926	−0.2 (−4.2, 4.7)	0.912	8.3 (4.5, 11.9)	0.000	617
HDL (mg%)	−0.7 (−2.8, 1.3)	0.467	0.3 (−1.7, 2.3)	0.782	−1.4 (3.3, 0.6)	0.166	−0.8 (−2.8, 1.1)	0.390	3.7 (2.1, 5.3)	0.000	617
Triglycerides (mg%)	−3.7 (−8.9, 1.5)	0.162	−6.2 (−11.8, −1.2)	0.017	1.7 (−5.9, 6.3)	0.511	−1.4 (−6.9, 4.1)	0.583	−7.7 (−11.8, −3.3)	0.000	617
Creatinine (mg%)	0.1 (−0.1, 0.1)	0.274	0.1 (−0.1, 0.1)	0.896	−0.1 (−0.1, 0.1)	0.752	−0.1 (−0.1, 0.1)	0.274	−0.2 (−0.2, −0.1)	0.000	617
Fasting insulin (mu/L)	−0.8 (−1.7, 0.4)	0.107	−0.9 (−1.7, 0.4)	0.072	0.6 (−0.3, 2.1)	0.209	−0.1 (−0.1, 2.1)	0.796	0.8 (−0.2, 1.4)	0.062	614
30-minute insulin (mu/L)	−16.9 (−30.2, −3.6)	0.013	−19.0 (−32.1, −6.0)	0.004	7.7 (−5.1, 20.5)	0.239	−9.2 (−22.0, 3.6)	0.160	7.2 (−3.3, 17.8)	0.179	581
2-hour insulin (mu/L)	−11.9 (−23.8, 0.5)	0.046	−9.9 (−20.8, 2.7)	0.092	1.6 (−7.4, 20.2)	0.778	−0.4 (−8.9, 15.6)	0.946	17.1 (6.2, 25.4)	0.000	577
C-reactive protein (mg/Dl)*	−0.2 (−0.5, 0.1)	0.144	−0.1 (−0.4, 0.2)	0.462	0.1 (−0.1, 0.4)	0.376	0.1 (−0.2, 0.3)	0.928	−0.2 (−0.4, 0.1)	0.066	610

F_0_, maternal generation; F_1_, offspring generation; CI, confidence interval; n, number.

^a^log transformed.

Models control for maternal age (continuous value, years) and parity (ref = 0). HDL, High-density lipoprotein.

**Figure 5:**
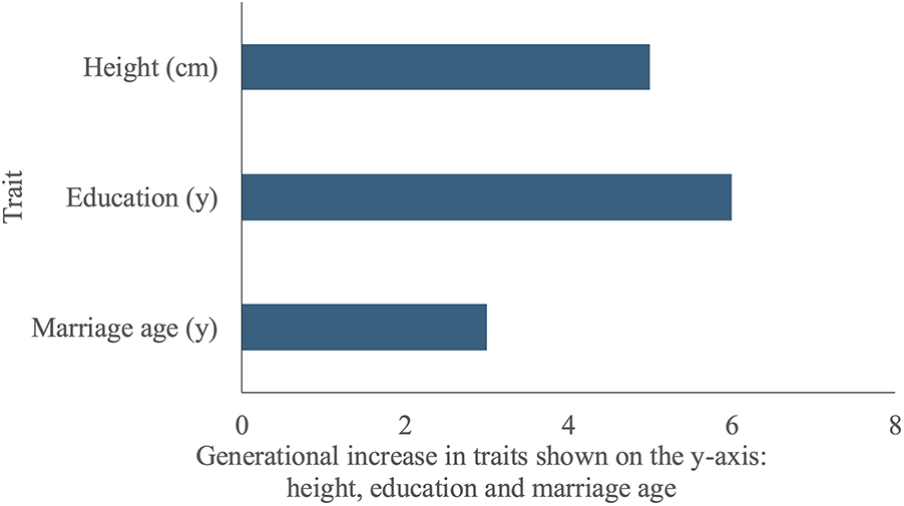
Secular trends, differences between F_1_ daughters’ and F_0_ mothers’ traits. The *y*-axis lists the traits being compared between F_1_ daughters and F_0_ mothers: height, education and marriage age. The *x*-axis indicates the difference in years of schooling completed, marriage age, and height (cm) between F_1_ daughters and F_0_ mothers. Daughters were taller, more educated and had married at a later age than their mothers.

Figure 5 describes secular trends differences between F_0_ mothers’ and F_1_ daughters’ outcomes. Maternal outcomes were measured at pre-pregnancy, daughters’ height and education at 18 years and daughter's age at marriage at 24 years. The mean height of daughters (157.0 cm, SD 5.6) was five cm greater than that of their mothers (152.0 cm, SD 4.7), suggesting an important secular trend in stature. The median years of schooling completed by daughters (12 years, Q1: 12, Q3: 13) was six years greater than their mothers (6 years, Q1: 1, Q3: 8). The median marriage age of daughters (21 years, Q1: 19.2, Q3: 25.9) was three years later than their mothers (18 years, Q1: 16, Q3: 19). Overall, 23% of daughters had married <19 years compared to 68% of mothers.

## Discussion

### Summary of results

This study quantified the associations of a mother's (F_0_) low maternal human capital with her own nutritional status and with offspring (F_1_) outcomes through the life-course, in a setting where early marriage, low educational attainment and maternal and child undernutrition have been common (83). All analyses controlled for socio-economic capital, and for sex when analysing F_1_ outcomes, allowing the magnitudes of effect for maternal human capital to be robustly quantified. Our study had six main findings.

First, low maternal human capital was associated with several markers of the mother's own undernutrition, including short stature, low body fatness, and micronutrient deficiency in folate. Contrary to these trends, low maternal human capital was associated with higher maternal vitamin B_12_ status, a finding discussed further below. Most of these markers have been linked with poor nutritional status in the offspring (4–7, 9–11, 96, 121). An additional notable finding was that for many outcomes, the magnitude of the deficit relative to the high-capital reference group was similar in both the low and mid maternal human capital groups. We suggest that the similar prevalence of early marriage in the low and mid human capital groups may explain their similar magnitudes of effect across many outcomes, which in turn would suggest that early marriage is a particularly important risk marker for undernutrition.

Second, low maternal human capital was associated with shorter gestation and markers of poor growth and undernutrition in offspring, though the magnitude of the effect varied by outcome and through development. There was no association of low human capital with height or weight *z*-scores at birth, although birth head girth was lower. Rather, more systematic associations of low human capital with poor F_1_ growth emerged in early post-natal life. Independent of both forms of maternal capital, there were sex differences in growth patterns, whereby girls showed greater deficits through childhood and adolescence. This may indicate delayed maturation in the girls of this cohort, compared to the UK reference data that generated the *z*-scores (126).

Third, low maternal human capital was associated with greater likelihood of offspring dropping out of secondary school, and of girls marrying and reproducing early. Notably, girls’ early reproduction was not preceded by earlier pubertal maturation, indicating that it was a lack of opportunity to accumulate human capital rather than accelerated physical development that precipitated early childbearing. Similar findings were previously reported in Brazil (56, 102). These results show how low human capital is transmitted to the next generation, particularly through the female line, whereby daughters of low human capital mothers are likely to accumulate low human capital themselves.

**Figure 6:**
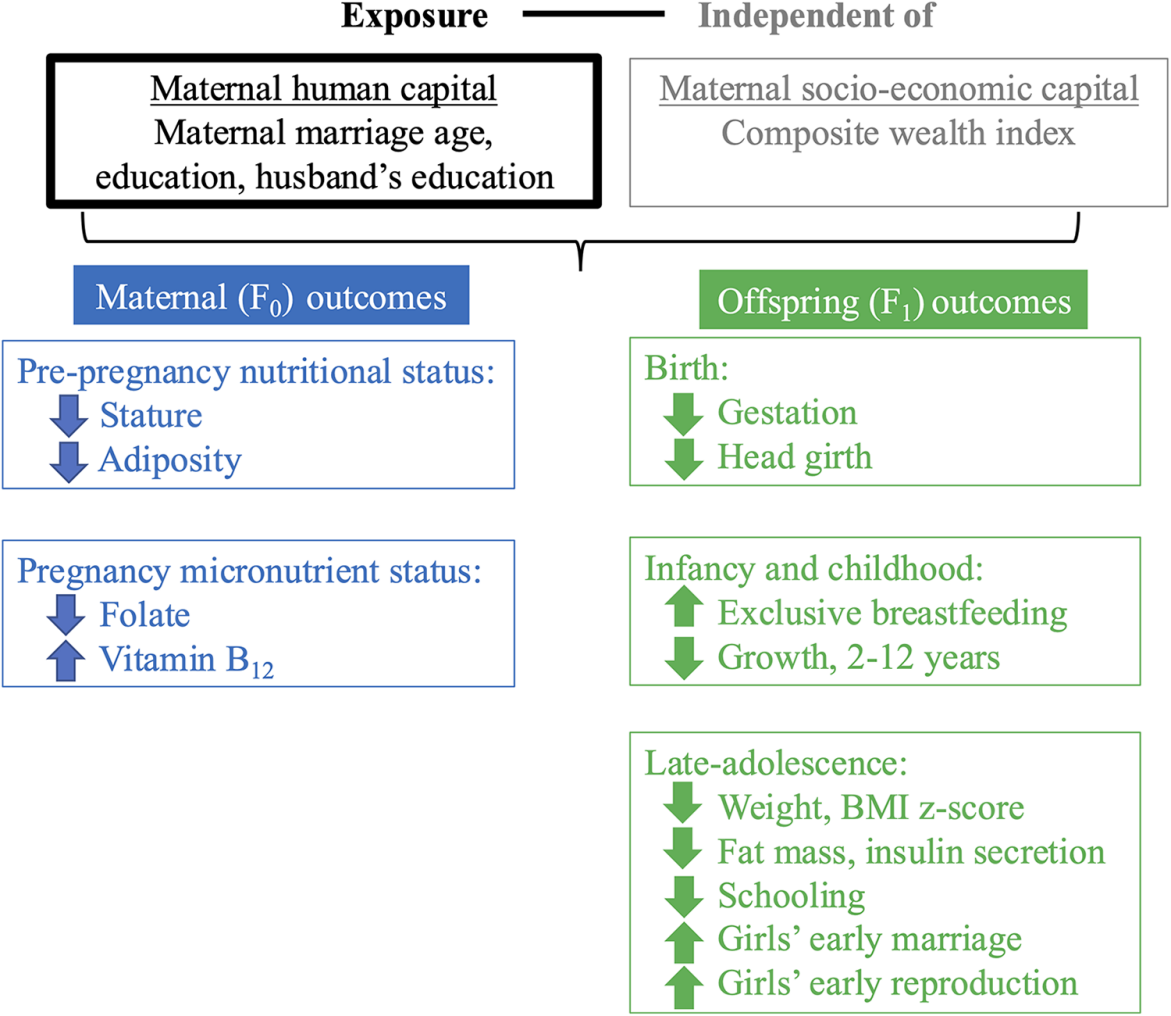
Summary of main findings. This figure reproduces the schematic diagram from Figure 2 to illustrate our findings. Independent of our secondary exposure (’socio-economic capital’, grey text), our primary exposure (low and mid F_0_ ‘maternal human capital’, black text) was associated with several F_0_ maternal outcomes at pre-pregnancy and pregnancy (blue text) and F_1_ offspring outcomes at birth, infancy/childhood and late-adolescence (green text). F_0_ refers to the maternal generation at baseline, and F_1_ to her offspring, or first generation. Downward facing arrows indicate that low maternal human capital was associated with lower values of the trait. Upward facing arrows indicate that low maternal human capital was associated with higher values of the trait.

Fourth, low maternal human capital showed few associations with offspring cardiometabolic risk. This indicates that the cycle of disadvantage currently remains restricted to the undernutrition component of malnutrition. These largely null results may be due partly to the offspring of low capital mothers having lower fat mass, a key driver on non-communicable disease risk, and partly to the young adult age of the assessment, as non-communicable disease risk accumulates through adult life. However, should the offspring gain weight in later adult life, they will likely display elevated cardiometabolic risk due to their low birth weight. The one outcome that did show an effect was 30-minute insulin (a marker of insulin secretion), which was lower in offspring of low and mid-capital mothers. This may indicate poorer beta-cell function and may indicate increased susceptibility to diabetes in the future, as the risk of diabetes accumulates with age. Figure 6, which is similar to our framework of analysis (Figure 2), summarises these four key findings.

Fifth, there were secular trends in nutritional status, education and marriage age between mothers and daughters. Daughters demonstrated substantial increases in mean stature, and in median educational attainment and marriage age. The proportion married early (<19 years) also decreased substantially across generations. Whilst these improvements are encouraging, the low capital mothers nevertheless had daughters with lower capital, indicating an intergenerational cycle of disadvantage.

Sixth, the associations of low human capital with maternal and offspring outcomes were much stronger than those for socio-economic capital, which were largely null. A potential explanation for these weak findings may be that a large proportion of our cohort (68%) was of higher caste affiliation and may therefore have had better economic resources. However, other studies have also found that greater household wealth does not improve child undernutrition; rather, the relevant factors ranged from food availability, access to quality healthcare, maternal nutrition, education and gender equality (33, 54, 57, 58). Moreover, the importance of maternal human capital may partly explain the lower than expected success of both economic growth conditional cash transfer programmes aiming to improve child malnutrition and delay girls’ marriage (41, 42, 134).

Collectively, our results offer support for our hypothesis of an over-arching cycle of disadvantage, whereby low-capital mothers demonstrated poor nutritional status, and had offspring demonstrating poor growth in early life, who went on to reproduce some of the low-capital traits of their mothers (low fat stores, lower education, and early marriage and early childbearing in girls). However, this cycle of disadvantage appeared to show very limited association with cardio-metabolic risk at a young age, though this may be partly because the young adult offspring are too young to have developed overt markers of non-communicable disease.

### Comparing results with previous research

Previous studies are consistent with our findings in reporting associations of low maternal education and early marriage with poorer maternal and child nutritional status (70, 88, 135, 136), and neurocognitive development in childhood (137). However, other than anaemia, to our knowledge, no other study has investigated the association of early marriage with women's micronutrient status and deficiency. One apparently contradictory finding was the higher levels of maternal vitamin B_12_ in the low- and mid-human capital mothers, compared to the high-capital mothers. However, this finding is similar to another study from India, where men with higher socio-economic status residing in middle-class urban areas had lower levels of vitamin B_12_ compared to men of lower socio-economic status residing in urban slums and rural areas (138). Higher vitamin B_12_ levels in disadvantaged groups may be explained by higher levels of microbes that are required to synthesise this micronutrient. In rural households of lower capital, this may be attributed to unhygienic characteristics of the water supply and the presence of livestock (138). Other studies have likewise found that processes that improve the potability of water, and that thereby reduce microbe content, are actually associated with increased risk of B_12_ deficiency (139, 140). Factors associated with maternal B_12_ during pregnancy may track to infants through transplacental transfer (56, 102, 141), however we did not evaluate offspring B_12_ status in this analysis.

Our finding of shorter gestation among lower human capital mothers has also been shown in other studies. For example, early marriage has been associated with preterm birth in Nepal, Bangladesh and other low-income contexts (84–86). The lack of association of low human capital with offspring birth size might be explained by maternal buffering during gestation, where mothers partially protect their offspring from their own undernutrition (51). After birth, however, the offspring of low-capital mothers grew poorly, as also reported in an analysis of maternal capital in a Brazilian cohort (56, 102).

Null results for socio-economic capital suggest that lack of access to, or cost of, supplementary foods are not the most important factors determining how long mothers breastfeed in this setting. The higher duration of breastfeeding among women with low human capital indicates that their poor nutritional status is not severe enough to impair their ability to breastfeed, but it may nevertheless affect the quality of the breastmilk, and hence might undermine healthy infant growth. Energy transfer through lactation may be more constrained, as shown in a study in rural and urban India (138). Low human capital mothers may also be less susceptible to norms that link supplementary foods with socio-economic social status. Studies from other populations in India have found that richer, higher educated women are less likely to breastfeed exclusively for 6 months (142–144), though the evidence is inconsistent (145).

The association of low maternal human capital with girls’ secondary school dropout, early marriage and reproduction indicates a composite intergenerational cycle of disadvantage. Other studies have found similar associations between mothers’ and daughters’ education and marriage age (97, 146). However, we found no evidence of poverty (measured at the time that F_0_ women were pregnant with F_1_ daughters) perpetuating lower education and early marriage across generations. In another analysis, wealth measured at different points in F_1_'s life-course, prior to marriage, was also not associated with early marriage (98). These results contrast with other studies (147, 148). Differences in the household where wealth was measured may explain these results. Previous studies have used women's marital household wealth, measured after marriage, as a proxy for their natal household's wealth (149, 150), to investigate whether poverty predicted their early marriage. In contrast, our study measured wealth in F_1_ girls’ natal household, before marriage. Although 77% of girls completed secondary school, 24% still married and reproduced early, suggesting that education may be leveraged to marry girls into a higher status family, rather than accessing other life opportunities (151, 152). Other factors beyond education may also matter for marital timing, as shown in a previous analysis of the PMNS cohort which found that independent of girls’ lower schooling, girls’ early marriage was associated with lower paternal education, being from a nuclear family, shorter gestation and girls’ poor infant weight gain (98).

### Limitations and strengths

Our study had some limitations. Data were missing on some variables, but the small magnitudes of difference between women and offspring with or missing these data are not expected to bias our results. Our sample size ranged from 651 to 442 depending on the outcome variable, and a larger study would be able to detect smaller differences between exposure and outcome variables. Other than education, we lacked data on the father's marriage age and nutritional status and therefore could not assess how his traits contributed to both maternal and offspring outcomes. Our marker of household wealth represented material assets and agricultural landholding and not the full range of financial flows (e.g., income and its differential access/use by different household members) relevant for our outcome variables. Our cohort was on average from the mid socio-economic group and higher caste affiliation, hence results may not be generalisable to the poorer and more socially disadvantaged groups. However, our cohort's profile reflects the rural population of Maharashtra state.

Our study also had some strengths. Our analysis used numerous markers of maternal nutritional status, including micronutrients, and detailed data on offspring nutritional status, growth and life trajectories from birth to early adulthood. Whilst we have noted the limitations of our wealth data (including that it was measured when F_0_ women were pregnant with F_1_ offspring), it was nevertheless measured in the girls’ natal household, and we could therefore examine its association with their education, marriage and reproduction age. Another analysis measured wealth at different points in F_1_'s life-course, prior to marriage, and found no association with F_1_ girls’ early marriage (98). Disentangling socio-economic and human capital can be challenging as proxy indicators such as education can plausibly contain elements of both. However, our PCA showed that maternal and husband's education predominantly mapped onto maternal marriage age, indicating human capital, and household wealth, caste and other socio-economic characteristics mapped onto socio-economic capital. Our associations are therefore robust, and our results are likely to be applicable to other rural South Asian societies where early marriage, childbearing and low maternal and child nutrition and education are prevalent.

## Conclusion

Overall, low maternal human capital, representing early marriage and lower education, was more important household socio economic capital for both maternal and child undernutrition. Moreover, these traits were to some extent replicated in the offspring, especially in daughters, demonstrating intergenerational transmission of low human capital. Our results have important implications for policy and programmes. Disentangling the independent role of socio-economic and maternal human capital in this cycle may help identify new opportunities for interventions to disrupt the cycle. Specifically, addressing the combination of low education, early marriage and their interplay with maternal undernutrition is crucial, because such a composite approach has the capacity to address several Sustainable Development Goals (1 to 5), relating to poverty, hunger, health and well-being and gender equality. To date, concerted efforts to address education and marriage age have had some success, though the pace of improvement has slowed following the COVID-19 pandemic (83, 153, 154). We argue that we now need both further research, to identify more effective strategies, and greater investment to scale up successful strategies.

## Data Availability

The datasets presented in this article are not readily available due to requirements of the local ethics committee and privacy concerns. Researchers who meet the criteria for accessing confidential data, and who understand the expectations of the local ethics committee and study participants can request data used in this analysis from the Principal Investigator, Professor CS Yajnik at csyajnik@gmail.com.
