# Supplementary material for: Like mother like daughter, the role of low human capital in intergenerational cycles of disadvantage: the Pune Maternal Nutrition Study
Source: Front Glob Womens Health. 2025 Jan 20;5:1174646. doi: 10.3389/fgwh.2024.1174646 (PMC11788374; doi:10.3389/fgwh.2024.1174646)
Supplement: Supplementary file 1 [file Table1.docx]

**Like mother like daughter, the role of low human capital in intergenerational cycles of disadvantage: the Pune Maternal Nutrition Study**

**Supplementary Table S1. Missing data (if missing >20 values)**

|  | **Missing**  ***n*=** | **Bias in missing data, tested for maternal age, pre-pregnancy stature and BMI** |
| --- | --- | --- |
| Exposure variables used to construct PCA capital groups missing on (F_0_) women | | |
| Maternal marriage age (years) | 33 | Younger mothers |
| Maternal education (years) | 42 | Younger mothers |
| Husband’s education (years) | 42 | Younger mothers |
| Socio-economic status (score) | 21 | No difference |
| Maternal outcome variables missing out of a cohort of 651 available for analysis | | |
|  | **Missing**  ***n*=** | **Bias in missing data, tested for maternal human capital and socio-economic capital groups, maternal age, pre-pregnancy stature and BMI** |
| Vitamin B_12_ (pmol/L), 28-weeks gestation | 98 | Low maternal human capital |
| Haemoglobin (gm/dL), 28-weeks gestation | 80 | Younger mothers, low maternal human capital |
| Red cell folate (ng/mL), 28-weeks gestation | 134 | No difference |
| Iron (microgm/L), 28-weeks gestation | 103 | Younger mothers |
| Serum vitamin C (micromol/l), 28-weeks gestation | 204 | Low maternal human capital, low socio-economic capital |
| Offspring (F_1_) outcome variables out of a cohort of 651 available for analysis | | |
| Placental weight (gm) | 81 | No difference |
| Birthweight (kg) | 34 | No difference |
| Height at 2 years (cm) | 30 | Higher BMI mothers |
| Weight at 2 years (kg) | 32 | Higher BMI mothers |
| BMI at 2 years (kg/m^2^) | 33 | Higher BMI mothers |
| Head circumference at 2 years (cm) | 29 | Higher BMI mothers |
| Pubertal timing (years) | 34 | No difference |
| Height at 18 years (cm) | 37 | Higher BMI mothers, low maternal human capital |
| Weight at 18 years (kg) | 37 | Higher BMI mothers, low maternal human capital |
| BMI at 18 years (kg/m^2^) | 37 | Higher BMI mothers, low maternal human capital |
| Head circumference at 18 years (cm) | 34 | Higher BMI mothers, low maternal human capital |
| Schooling completed at 18 years (years) | 40 | Higher BMI mothers, low maternal human capital |
| F_1_ biomarkers at 18 years | | |
| Fat mass (kg) | 40 | No difference |
| Fat free mass (kg) | 40 | No difference |
| Systolic blood pressure (mmHg) | 34 | Higher BMI mothers, low maternal human capital |
| Diastolic blood pressure (mmHg) | 34 | Higher BMI mothers, low maternal human capital |
| Fasting glucose (mg%) | 34 | Higher BMI mothers, low maternal human capital |
| 30-minute glucose (mg%) | 68 | Low maternal human capital |
| 2-hour glucose (mg%) | 71 | Low maternal human capital |
| Cholesterol (mg%) | 34 | Higher BMI mothers, low maternal human capital |
| HDL (mg%) | 34 | Higher BMI mothers, low maternal human capital |
| Triglycerides (mg%) | 34 | Higher BMI mothers, low maternal human capital |
| Creatinine (mg%) | 34 | Higher BMI mothers, low maternal human capital |
| Fasting insulin (mu/L) | 36 | Higher BMI mothers, low maternal human capital |
| Insulin secretion (mu/L) | 70 | Low maternal human capital |
| 2-hour insulin (mu/L) | 74 | No difference |
| C-reactive protein (mg/dL) | 36 | Higher BMI mothers, low maternal human capital |

*n*, number. F_0_, maternal generation. F_1_, offspring generation.
