# Supplementary material for: Like mother like daughter, the role of low human capital in intergenerational cycles of disadvantage: the Pune Maternal Nutrition Study
Source: Front Glob Womens Health. 2025 Jan 20;5:1174646. doi: 10.3389/fgwh.2024.1174646 (PMC11788374; doi:10.3389/fgwh.2024.1174646)
Supplement: Supplementary file 2 [file Table2.docx]

**Like mother like daughter, the role of low human capital in intergenerational cycles of disadvantage: the Pune Maternal Nutrition Study**

**Supplementary Table S2. Description of composite F_0_ maternal human and socio-economic capital groups (*n*=659)**

| **PCA 1:**  **Maternal human capital** | **Maternal marriage age (years)** | **Maternal education**  **(years)** | **Husband’s education**  **(years)** |
| --- | --- | --- | --- |
|  | **Median (Q1,Q3)** | **Median (Q1,Q3)** | **Median (Q1,Q3)** |
| Low | 17 (15, 18) | 0 (0, 4) | 4 (0, 7) |
| Mid | 17 (16, 19) | 7 (5, 7) | 9 (7, 10) |
| High | 19 (18, 20) | 9 (7, 10) | 10 (10, 12) |
| **PCA 2:**  **Socio-economic capital** | **Socio-economic status score^1^** | n/a | |
| Low | 21 (17, 23) |  |  |
| Mid | 27 (26, 28) |  |  |
| High | 33 (31, 36) |  |  |

F_0_, maternal generation. Q1, 25^th^ percentile. Q3, 75^th^ percentile. ^1^Asset score ranges from 6 to 47. n/a, not applicable.
