# Supplementary material for: Like mother like daughter, the role of low human capital in intergenerational cycles of disadvantage: the Pune Maternal Nutrition Study
Source: Front Glob Womens Health. 2025 Jan 20;5:1174646. doi: 10.3389/fgwh.2024.1174646 (PMC11788374; doi:10.3389/fgwh.2024.1174646)
Supplement: Supplementary file 3 [file Table3.docx]

**Like mother like daughter, the role of low human capital in intergenerational cycles of disadvantage: the Pune Maternal Nutrition Study**

**Supplementary Table S3. Differences in F_1_ size and nutritional status (z-scores) from birth to 18 years and pubertal age, stratified by sex**

|  | **Full sample** | | **Girls** | | **Boys** | | **Difference^1^** | |
| --- | --- | --- | --- | --- | --- | --- | --- | --- |
| **F_1_ outcomes** | ***n*** | **Mean (SD)** | ***n*** | **Mean (SD)** | ***n*** | **Mean (SD)** | **∆ (95% CI)** | ***p*-value** |
| Birthweight (kg) | 617 | 2.6 (0.4) | 293 | 2.5 (0.4) | 324 | 2.7 (0.4) | -0.1 (-0.2, -0.1) | 0.000 |
| Birth length (cm) | 641 | 47.5 (2.3) | 306 | 47.1 (2.0) | 335 | 47.8 (2.4) | -0.7 (-1.0, -0.3) | 0.000 |
| Birth head circumference (cm) | 641 | 32.9 (1.4) | 306 | 32.6 (1.4) | 335 | 33.2 (1.4) | -0.6 (-0.8, -0.4) | 0.000 |
| WAZ0 | 617 | -1.6 (0.9) | 293 | -1.6 (0.9) | 324 | -1.5 (0.8) | -0.2 (-0.3, -0.1) | 0.028 |
| HAZ0 | 641 | -1.1 (0.9) | 306 | -1.1 (0.9) | 335 | -1.0 (0.9) | -0.1 (-0.3, 0.1) | 0.132 |
| HCAZ0 | 641 | -1.0 (1.0) | 306 | -1.0 (1.1) | 335 | -1.0 (0.9) | -0.1 (-0.2, 0.1) | 0.351 |
| WAZ2 | 619 | -1.9 (1.0) | 294 | -1.8 (1.0) | 325 | -1.9 (0.9) | 0.1 (-0.1, 0.2) | 0.894 |
| HAZ2 | 621 | -1.7 (1.1) | 293 | -1.7 (1.0) | 328 | -1.7 (1.1) | 0.1 (-0.1, 0.2) | 0.586 |
| BAZ2 | 618 | -1.2 (1.0) | 293 | -1.1 (1.0) | 325 | -1.2 (0.9) | 0.1 (-0.1, 0.2) | 0.896 |
| HCAZ2 | 622 | -1.5 (0.9) | 294 | -1.5 (0.8) | 328 | -1.4 (0.9) | -0.1 (-0.3, 0.1) | 0.103 |
| WAZ6 | 649 | -2.2 (1.0) | 310 | -2.2 (0.9) | 339 | -2.2 (1.0) | 0.1 (-0.1, 0.2) | 0.904 |
| HAZ6 | 649 | -1.4 (0.9) | 310 | -1.4 (0.8) | 339 | -1.4 (0.9) | -0.1 (-0.2, 0.1) | 0.401 |
| BAZ6 | 649 | -1.9 (0.9) | 310 | -1.8 (0.8) | 339 | -1.9 (0.9) | 0.1 (-0.1, 0.2) | 0.418 |
| HCAZ6 | 649 | -3.0 (1.1) | 310 | -3.4 (1.2) | 339 | -2.7 (0.9) | -0.7 (-0.9, -0.5) | 0.000 |
| WAZ12 | 651 | -1.7 (1.1) | 310 | -1.7 (1.1) | 341 | -1.7 (1.1) | 0.1 (-0.2, 0.2) | 0.972 |
| HAZ12 | 651 | -1.1 (0.9) | 310 | -1.0 (0.9) | 341 | -1.1 (0.9) | 0.1 (-0.1, 0.2) | 0.214 |
| BAZ12 | 651 | -1.7 (1.2) | 310 | -1.9 (1.3) | 341 | -1.6 (1.1) | 0.1 (-0.4, -0.1) | 0.010 |
| HCAZ12 | 645 | -2.3 (1.1) | 305 | -2.4 (1.3) | 340 | -2.1 (0.9) | -0.3 (-0.5, -0.1) | 0.000 |
| Girls’ age at menarche (years) | n/a | n/a | 305 | 13.4 (1.1) | n/a | n/a | n/a | n/a |
| Age at puberty (years) | 586 | 11.5 (0.9) | 296 | 11.6 (0.9) | 321 | 11.5 (1.0) | 0.1 (-0.1, 0.3) | 0.153 |
| WAZ18 | 616 | -1.6 (1.4) | 284 | -1.8 (1.3) | 332 | -1.5 (1.5) | -0.2 (-0.5, 0.1) | 0.025 |
| HAZ18 | 616 | -1.1 (0.9) | 284 | -1.1 (0.9) | 332 | -1.1 (0.9) | -0.1 (-0.2, 0.1) | 0.847 |
| BAZ18 | 616 | -1.1 (1.4) | 284 | -1.2 (1.3) | 332 | -1.0 (1.5) | -0.3 (-0.5, -0.1) | 0.032 |
| HCAZ18 | 615 | -2.0 (0.9) | 284 | -2.0 (0.9) | 331 | -2.1 (0.9) | 0.1 (-0.1, 0.2) | 0.396 |

F_1_, offspring generation. WAZ, weight z-score, HAZ, height z-score, BAZ, BMI z-score, HCAZ, head circumference z-score. *n*, number. SD, standard deviation. ∆, difference between girls and boys. CI, confidence interval. n/a, not applicable. ^1^Independent samples *t*-test.
