# Supplementary material for: Like mother like daughter, the role of low human capital in intergenerational cycles of disadvantage: the Pune Maternal Nutrition Study
Source: Front Glob Womens Health. 2025 Jan 20;5:1174646. doi: 10.3389/fgwh.2024.1174646 (PMC11788374; doi:10.3389/fgwh.2024.1174646)
Supplement: Supplementary file 4 [file Table4.docx]

**Like mother like daughter, the role of low human capital in intergenerational cycles of disadvantage: the Pune Maternal Nutrition Study**

**Supplementary Table S4. OLS linear regression of F_0_ maternal human capital and F_1_ size and nutritional status (z-scores), birth to 18 years**

|  | **F_0_ exposures** | | | | | | | | **F_1_ outcome** | |  |
| --- | --- | --- | --- | --- | --- | --- | --- | --- | --- | --- | --- |
|  | **PCA 1: Maternal human capital (ref: high capital)** | | | | **PCA 2: Socio-economic capital (ref: high capital)** | | | | **Offspring sex (ref: boys)** | |  |
|  | **Low capital** | | **Mid capital** | | **Low capital** | | **Mid capital** | | **Girl** | |  |
| **F_1_ outcomes** | **ß (95% CI)** | ***p*-value** | **ß (95% CI)** | ***p*-value** | **ß (95% CI)** | ***p*-value** | **ß (95% CI)** | ***p*-value** | **ß (95% CI)** | ***p*-value** | ***n*** |
| WAZ0 | 0.1 (-0.2, 0.2) | 0.923 | 0.1 (-0.1, 0.2) | 0.338 | -0.1 (-0.2, 0.1) | 0.533 | -0.2 (-0.4, -0.1) | 0.006 | -0.1 (-0.3, -0.1) | 0.031 | 617 |
| HAZ0 | 0.1 (-0.1, 0.3) | 0.227 | 0.1 (-0.1, 0.3) | 0.343 | -0.1 (-0.3, 0.1) | 0.183 | -0.2 (-0.3, 0.1) | 0.068 | -0.1 (-0.3, 0.1) | 0.110 | 641 |
| HCAZ0 | -0.1 (-0.3, 0.1) | 0.272 | -0.1 (-0.3, 0.1) | 0.350 | 0.1 (-0.2, 0.1) | 0.690 | -0.1 (-0.3, 0.1) | 0.322 | -0.1 (-0.2, 0.1) | 0.461 | 641 |
| WAZ2 | -0.3 (-0.5, -0.1) | 0.006 | -0.1 (-0.3, 0.1) | 0.481 | -0.2 (-0.5, -0.1) | 0.002 | -0.2 (-0.4, 0.1) | 0.073 | 0.1 (-0.1, 0.2) | 0.778 | 619 |
| HAZ2 | -0.3 (-0.5, -0.1) | 0.009 | -0.1 (-0.2, 0.2) | 0.849 | -0.2 (-0.4, 0.1) | 0.063 | -0.2 (-0.4, 0.1) | 0.076 | 0.1 (-0.1, 0.2) | 0.454 | 621 |
| BAZ2 | -0.1 (-0.3, 0.1) | 0.328 | -0.1 (-0.3, 0.1) | 0.415 | -0.2 (-0.4, -0.1) | 0.028 | -0.1 (-0.3, 0.1) | 0.465 | 0.1 (-0.1, 0.2) | 0.920 | 618 |
| HCAZ2 | -0.2 (-0.4, -0.1) | 0.011 | -0.1 (-0.2, 0.1) | 0.781 | -0.1 (-0.3, 0.1) | 0.158 | -0.1 (-0.2, 0.1) | 0.579 | -0.1 -0.2, 0.1) | 0.151 | 622 |
| WAZ6 | -0.3 (-0.5, -0.1) | 0.006 | -0.1 (-0.2, 0.1) | 0.717 | -0.2 (-0.4, -0.1) | 0.046 | -0.2 (-0.4, -0.1) | 0.010 | 0.1 (-0.1, 0.2) | 0.726 | 649 |
| HAZ6 | -0.2 (-0.4, -0.1) | 0.036 | -0.1 (-0.1, 0.2) | 0.567 | -0.3 (-0.4, 0.1) | 0.002 | -0.2 (-0.4, -0.1) | 0.014 | -0.1 (-0.2, 0.1) | 0.444 | 649 |
| BAZ6 | -0.2 (-0.4, -0.1) | 0.045 | -0.2 (-0.3, 0.1) | 0.146 | 0.1 (-0.1, 0.2) | 0.619 | -0.1 (-0.3, -0.1) | 0.146 | 0.1 (-0.1, 0.2) | 0.274 | 649 |
| HCAZ6 | -0.1 (-0.3, 0.1) | 0.323 | 0.0 (-0.2, 0.2) | 0.998 | -0.2 (-0.4, 0.1) | 0.080 | -0.2 (-0.4, 0.1) | 0.092 | -0.7 (-0.9, -0.5) | 0.000 | 649 |
| WAZ12 | -0.3 (-0.5, -0.1) | 0.026 | -0.1 (-03, 0.1) | 0.383 | -0.1 (-0.2, 0.2) | 0.876 | -02 (-0.4, 0.1) | 0.066 | 0.1 (-0.2, 0.2) | 0.805 | 651 |
| HAZ12 | -0.1 (-0.3, 0.0) | 0.127 | 0.1 (-0.2, 0.2) | 0.768 | -0.2 (-0.4, -0.1) | 0.033 | -0.2 (-0.4, -0.1) | 0.023 | 0.1 (-0.1, 0.2) | 0.179 | 651 |
| BAZ12 | -0.2 (-0.4, 0.1) | 0.092 | -0.2 (-0.4, 0.1) | 0.102 | 0.2 (-0.1, 0.4) | 0.168 | -0.1 (-0.4, 0.1) | 0.276 | -0.2 (-0.4, -0.1) | 0.022 | 651 |
| HCAZ12 | -0.1 (-0.3, 0.1) | 0.524 | -0.1 (-0.3, 0.2) | 0.939 | 0.1 (-0.2, 0.2) | 0.789 | -0.1 (-0.3, 0.1) | 0.306 | -0.3 (-0.5, -0.1) | 0.000 | 645 |
| WAZ18 | -0.3 (-0.6, -0.1) | 0.025 | -0.2 (-0.5, 0.1) | 0.292 | 0.1 (-0.3, 0.2) | 0.928 | -0.2 (-0.5, 0.1) | 0.176 | -0.2 (-0.4, -0.1) | 0.046 | 616 |
| HAZ18 | -0.1 (-0.3, 0.1) | 0.480 | 0.1 (-0.1, 0.3) | 0.175 | -0.2 (-0.4, -0.1) | 0.026 | -0.2 (-0.4, 0.1) | 0.054 | -0.1 (-0.2, 0.1) | 0.799 | 616 |
| BAZ18 | -0.3 (-0.6, -0.1) | 0.039 | -0.3 (-0.6, 0.1) | 0.069 | 0.2 (-0.1, 0.4) | 0.273 | -0.1 (-0.4, 0.2) | 0.509 | -0.2 (-0.5, -0.1) | 0.061 | 616 |
| HCAZ18 | -0.1 (-0.3, 0.1) | 0.468 | -0.1 (-0.2, 0.2) | 0.722 | -0.1 (-0.2, 0.1) | 0.289 | -0.1 (-0.3, 0.1) | 0.432 | 0.1 (-0.1, 0.2) | 0.213 | 615 |

F_0_, maternal generation. F_1_, offspring generation. WAZ, weight z-score, HAZ, height z-score, BAZ, BMI z-score, HCAZ, head circumference z-score. CI, confidence interval. n, number. Models control for maternal age (continuous value, years) and parity (ref=0).
