# Supplementary material for: Like mother like daughter, the role of low human capital in intergenerational cycles of disadvantage: the Pune Maternal Nutrition Study
Source: Front Glob Womens Health. 2025 Jan 20;5:1174646. doi: 10.3389/fgwh.2024.1174646 (PMC11788374; doi:10.3389/fgwh.2024.1174646)
Supplement: Supplementary file 5 [file Table5.docx]

**Like mother like daughter, the role of low human capital in intergenerational cycles of disadvantage: the Pune Maternal Nutrition Study**

**Supplementary Table S5. Adjusted multivariable logistic regression of F_0_ maternal human capital and F_1_ outcomes**

|  | **F_0_ exposures** | | | | | | | | **F_1_ outcome** | |  |  |
| --- | --- | --- | --- | --- | --- | --- | --- | --- | --- | --- | --- | --- |
|  | **PCA 1: Maternal human capital**  **(ref: high capital)** | | | | **PCA 2: Socio-economic capital**  **(ref: high capital)** | | | | **Offspring sex**  **(ref: boys)** | |  |  |
|  | **Low capital** | | **Mid capital** | | **Low capital** | | **Mid capital** | | **Girl** | |  |  |
| **F_1_ outcomes** | **aOR**  **(95% CI)** | ***p*-value** | **aOR**  **(95% CI)** | ***p*-value** | **aOR**  **(95% CI)** | ***p*-value** | **aOR**  **(95% CI)** | ***p*-value** | **aOR**  **(95% CI)** | ***p*-value** | ***n*** | **NK** |
| Pre-term birth <37 weeks | 1.1 (0.5, 2.2) | 0.802 | 1.4 (0.7, 2.8) | 0.303 | 1.3 (0.7, 2.5) | 0.383 | 1.0 (0.5, 2.0) | 0.924 | 1.1 (0.6, 1.8) | 0.855 | 651 | 0.034 |
| Low birthweight <250 kg | 1.1 (0.7, 1.7) | 0.661 | 0.8 (0.5, 1.3) | 0.445 | 1.3 (0.8, 2.0) | 0.250 | 1.6 (1.0, 2.5) | 0.032 | 2.1 (1.5, 3.0) | 0.000 | 617 | 0.072 |
| Exclusive breastfeeding  <6 months | 0.4 (0.2, 0.6) | 0.000 | 0.6 (0.4, 0.9) | 0.036 | 1.0 (0.7, 1.6) | 0.933 | 1.1 (0.7, 1.7) | 0.684 | 0.8 (0.6, 1.2) | 0.246 | 647 | 0.078 |
| Secondary school drop-out <12^th^ standard | 4.2 (2.4, 7.4) | 0.000 | 2.0 (1.0, 3.7) | 0.025 | 2.0 (1.2, 3.4) | 0.012 | 1.7 (0.9, 2.8) | 0.063 | 1.5 (0.9, 2.2) | 0.080 | 611 | 0.132 |
| Pre-diabetic at 18 years | 1.1 (0.6, 1.9) | 0.700 | 1.3 (0.7, 2.2) | 0.372 | 0.9 (0.6, 1.7) | 0.918 | 0.9 (0.6, 1.6) | 0.877 | 0.4 (0.3, 0.6) | 0.000 | 426 | 0.058 |
| Girls’ early marriage <19 years | 2.7 (1.3, 5.5) | 0.005 | 0.8 (0.3, 1.6) | 0.609 | 1.5 (0.2, 1.1) | 0.250 | 1.3 (0.4, 2.0) | 0.418 | n/a | n/a | 310 | 0.108 |
| Girls’ early reproduction <20 years | 2.2 (0.9, 5.2) | 0.085 | 0.6 (0.2, 1.6) | 0.309 | 2.2 (0.9, 5.4) | 0.094 | 1.1 (0.5, 2.6) | 0.769 | n/a | n/a | 189 | 0.180 |

F_0_, maternal generation. F_1_, offspring generation. aOR, adjusted Odds Ratio. CI, confidence interval. *n*, number. NK, NagelKerke, pseudo R^2^. n/a, not applicable. Models control for maternal age (continuous value, years) and parity (ref=0).
