# Supplementary figures and images for: Like mother like daughter, the role of low human capital in intergenerational cycles of disadvantage: the Pune Maternal Nutrition Study
Source: Front Glob Womens Health. 2025 Jan 20;5:1174646. doi: 10.3389/fgwh.2024.1174646 (PMC11788374; doi:10.3389/fgwh.2024.1174646)

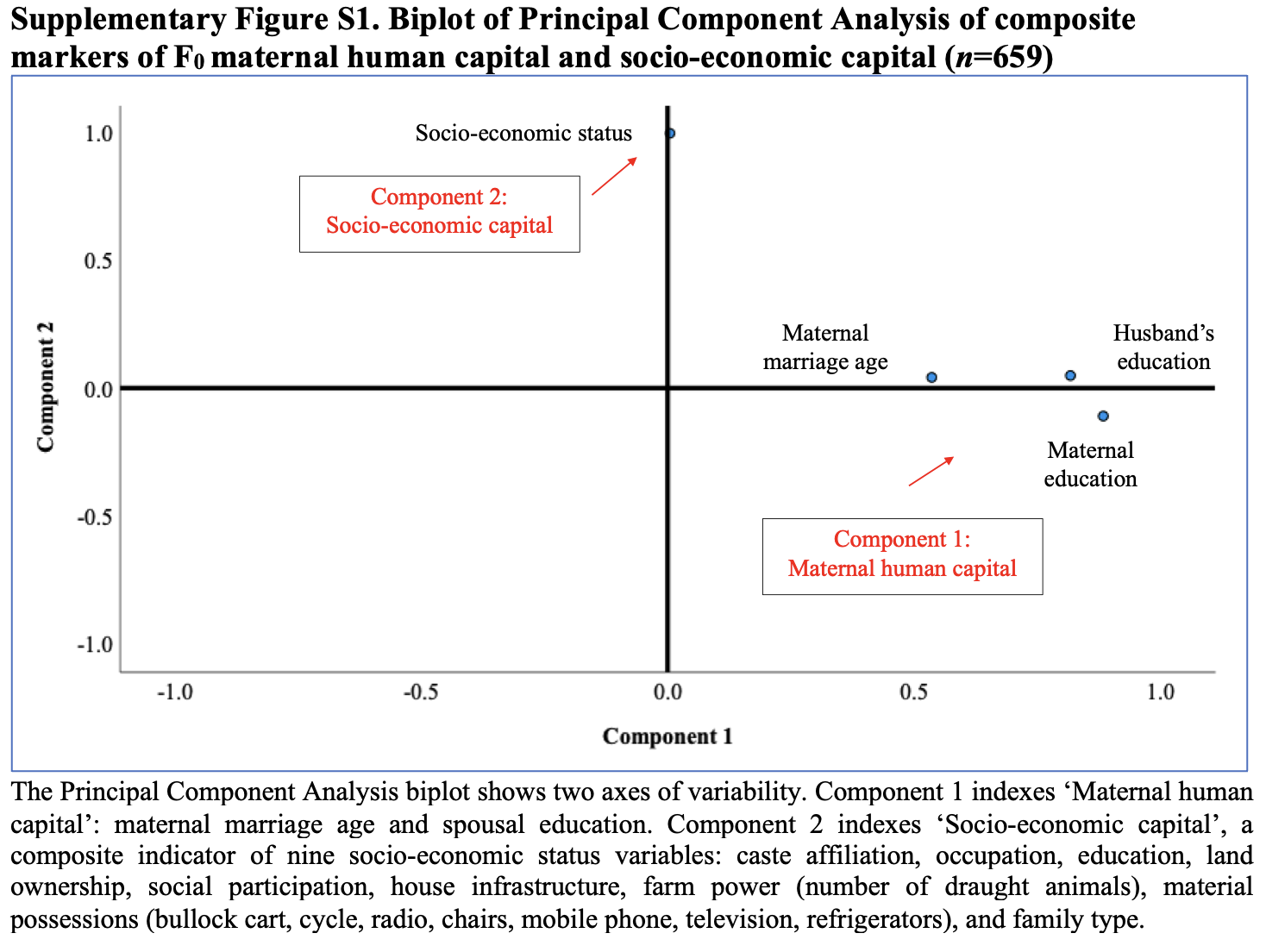

Supplement: Supplementary file 6 [file Image1.tiff]
